# Cooking at Home and Adherence to the Mediterranean Diet During the COVID-19 Confinement: The Experience From the Croatian COVIDiet Study

**DOI:** 10.3389/fnut.2021.617721

**Published:** 2021-03-31

**Authors:** Danijela Pfeifer, Josip Rešetar, Jasenka Gajdoš Kljusurić, Ines Panjkota Krbavčić, Darija Vranešić Bender, Celia Rodríguez-Pérez, María Dolores Ruíz-López, Zvonimir Šatalić

**Affiliations:** ^1^Faculty of Food Technology and Biotechnology, University of Zagreb, Zagreb, Croatia; ^2^Faculty of Pharmacy and Biochemistry, University of Zagreb, Zagreb, Croatia; ^3^Clinical Unit of Clinical Nutrition, Department of Internal Medicine, University Hospital Centre Zagreb, Zagreb, Croatia; ^4^Department of Nutrition and Food Science, University of Granada, Melilla, Spain; ^5^Biomedical Research Centre, Institute of Nutrition and Food Technology (INYTA) ‘JoséMataix’, University of Granada, Granada, Spain; ^6^Biosanitary Research Institute of Granada, Granada, Spain; ^7^Department of Nutrition and Food Science, University of Granada, Granada, Spain

**Keywords:** body mass index, cooking, COVID-19 confinement, mediterranean diet, questionnaire, SARS-CoV-2

## Abstract

**Introduction:** The primary aims of this study were to evaluate the changes in dietary behavior among the Croatian adult population during the COVID-19 outbreak and to explore the impact of confinement on cooking habits.

**Methods:** The study was based on results from COVIDiet_Int cross-sectional study—a part of COVIDiet project (NCT04449731). A self-administered online questionnaire was used to assess the frequency of food consumption, eating habits, and sociodemographic information. A total number of 4,281 participants (80.5% females and 19.4% males) completed the questionnaire.

**Results:** The Mediterranean Diet Adherence Screener (MEDAS) score before the confinement was 5.02 ± 1.97, while during the confinement, the MEDAS score increased to 5.85 ± 2.04. Participants who had higher adherence to the Mediterranean diet (MedDiet) during the confinement were mostly females (88.8%), aged between 20 and 50 years, with the highest level of education (66.3%) and normal BMI (70.6%). The majority of participants maintained their dietary behavior as it was before COVID-19 confinement, while 36.9% decreased their physical activity. Participants with higher MEDAS score were more eager to increase their physical activity. Additionally, higher median values of MEDAS score were noted for participants with body mass index values below 24.9 kg/m^2^ (6.0 vs. 5.0 for participants with BMI above 25 kg/m^2^). Participants in all residence places increased their cooking frequency during the confinement (53.8%), which was associated with an increase in vegetables, legumes, as well as fish and seafood consumption.

**Conclusions:** According to our findings, Croatian adults exhibited medium adherence to the MedDiet during the COVID-19 confinement. The results suggest that cooking frequency could be positively associated with overall dietary quality, which is of utmost importance in these demanding times.

## Introduction

The novel coronavirus disease, COVID-19, caused by the Severe Acute Respiratory Syndrome Coronavirus-2 (SARS-CoV-2), emerged in late December 2019, in China, and since then, it has been one of the greatest public health crises the world has ever faced. As a result of its rapid spread, high infectivity, and relatively high mortality, the World Health Organization (WHO) has declared COVID-19 a pandemic on March 12, 2020 (1). To date, on January 29, more than 100 million cases of COVID-19 have been confirmed and 2,176,159 deaths reported globally (2). In Croatia, up until January 29, the number of COVID-19-positive people was 231,539, while 4,972 people passed away (3).

Although vaccination against SARS-CoV-2 started at the end of the year 2020, the main strategy to attenuate the rate of infection includes community-based non-pharmaceutical interventions (NPIs) such as social distancing, restricting public events, case-based isolation, and confinement (4). Croatia follows a similar strategy pattern—a national confinement policy was implemented; public events and many economic activities were stopped, and schools and universities were closed on March 19. After almost 5 weeks of confinement, due to a beneficial epidemiological situation, Croatia started to reactivate its economic and social life in three phases—the first phase, when some business entities were allowed to work, began on April 27; the second phase, when playgrounds and outdoor sports facilities were opened, began on May 4; the third phase, when shopping centers, schools, and kindergartens were reopened, began on May 11 (5).

Even though NPIs, like confinement and isolation, can mitigate and control the rate of COVID-19 spread (6), they can also lead to boredom, mental distress, emotional imbalance, change in eating behavior, weight gain, and higher risk of cardiovascular disease (7). Staying at home and working from home can result in boredom, which is positively associated with higher energy intake (8). However, more free time during confinement could also bring about positive eating behavior and habits—more time spent cooking, family meals, and better meal planning. Additionally, closure of gyms, recreation facilities, and prohibition of sports activities could result in negative well-being, low-energy expenditure, and cardiometabolic health impairment (9). Maintaining adequate energy balance is essential in the time of COVID-19 pandemic due to a positive link found between severe COVID-19 cases and obesity (10, 11).

Optimal nutritional status has a pivotal role in immune system functioning and could help mitigate the impact of seasonal and emerging viral infections (12). There are already several papers supporting nutraceuticals, probiotics, and supplements alongside a well-balanced diet as a method of managing COVID-19 (13, 14). Bousquet et al. (15) suggest that certain foods may reduce angiotensin-converting enzyme 2 (ACE2) activity, which is, beside multiple physiological roles, a SARS-CoV-2 receptor. Considering the importance of nutrition during COVID-19 pandemic, the European Federation of the Associations of Dietitians (16) as well as the WHO (17) have made public health nutrition advice and guidelines for maintaining an optimal nutrition status. Furthermore, Muscogiuri et al. (18) published a perspective on nutritional recommendations during the COVID-19 confinement. All the recommendations promote nutrients, foods, and dietary patterns that are included in a Mediterranean diet (MedDiet), which is considered healthy and anti-inflammatory (19).

In contrast to other popular diets, the MedDiet has suggestive evidence of an improvement in weight, BMI, total cholesterol, glucose, and blood pressure (20). Obesity, in terms of COVID-19, is associated with a higher risk for hospitalization and higher death rates (21), which also highlights the current necessity for proper nutrition. The MedDiet favorable effects, both therapeutic and preventive, are proposed in detail elsewhere (22, 23). Moreover, a higher adherence to the MedDiet during the COVID-19 crisis is suggested not only to combat the COVID-19-related complications but also to lower the risk of chronic diseases (24). However, due to shortages of some foods in grocery stores during confinement and financial concerns, it is not very certain if people could fully follow the MedDiet guidelines.

The aims of this study were (i) to evaluate the changes in dietary behavior among the Croatian adult population, (ii) to investigate adherence to the MedDiet, (iii) to examine the availability of certain foods, (iv) to determine differences in adherence to MedDiet among two BMI groups (<25 and >25 kg/m^2^), and (v) to explore the cooking habits during the COVID-19 outbreak.

## Materials and Methods

### Study Design and Participants

COVIDiet_Int is a cross-sectional study and a part of a COVIDiet project, which included Croatian adults and had no exclusion criteria other than age. A study has been conducted in accordance with the World Medical Association's Declaration of Helsinki, approved by the Research Ethics Committee of the University of Granada (1526/CEIH/2020), and registered as a clinical trial in a public trials registry (NCT04449731). Since the data were collected anonymously by the use of an online questionnaire, without including personal data, no informed consent was required. Despite that, all the participants were informed about the study objectives and asked for a permission before filling out a questionnaire. The questionnaire was opened from April 7 until May 4. Data were collected through snowball sampling.

### Data Collecting—Questionnaire

A self-administered online questionnaire, created using the Google Forms tool, was used to assess the frequency of food consumption, eating habits, and sociodemographic information. Sociodemographic characteristics included age, gender, place of residence, country region, children in care, and education and training qualifications. The assessment of food consumption frequency included selected foods related to the MedDiet pattern through MEDAS-validated questionnaire (24, 25). For the purpose of this study, respondents were asked to estimate the consumed quantity of (i) olive oil (0–1.9, 2–3.9, or >4 tablespoons (1 tablespoon = 13.5 g)/day), (ii) vegetables (0–0.9, 1–1.9, or >2 servings (1 serving = 200 g)/day), (iii) fruit (0–0.9, 1–2.9, or >3 pieces/day), (iv) red meat (0–0.9 or >1 serving (1 serving = 100–150 g)/day), (v) butter, margarine, or cream (0–0.9 or >1 serving (1 serving = 12 g)/day), (vi) carbonated and/or sugary beverages (0–0.9 or >1 time/day), (vii) wine (0–2.9, 3–6.9, or >7 cups (1 cup = 100 ml)/week or never), (viii) legumes (0–0.9, 1–2.9, or >3 servings (1 serving = 150 g)/week), (ix) fish and seafood (0–0.9, 1–2.9, or >3 servings (1 serving = 100–150 g for fish or 200 g for seafood)/week), (x) commercial pastries (0–1.9 or >2 times/week), (xi) nuts (0–0.9, 1–2.9, or >3 servings (1 serving = 30 g)/week), and (xii) cooked vegetables, pasta, rice, or dishes seasoned with tomato, garlic, onion, or leek sauce made over low heat with olive oil (0–0.9, 1–1.9, or >2 times/week). After that, eating habits assessment contained 21 questions aimed at investigating the change in dietary habits during COVID-19 confinement, for example, frequency of snacking, consuming fried food, type of oil used when frying, difficulties in finding certain food, among others. Therefore, participants provided the information whether they had perceived any change related to the nutrition during the COVID-19 confinement in comparison with the situation before that. Additionally, they were also asked to provide information about the change in physical activity as well as in body weight during the confinement. Based on the values that the respondents self-assessed, the body mass index (BMI) was calculated as the ratio of the body weight [in (kg)] and the square of the body height [in (m)]. A more comprehensive insight into the questionnaire description as well as the full form of questionnaire is available in a paper published by Rodríguez-Pérez et al. (24).

### Adherence Based on Mediterranean Diet Adherence Screener

The MedDiet adherence was measured by the Mediterranean Diet Adherence Screener, the MEDAS (25). The MEDAS consists of 14 questions where 12 aforementioned questions are related to food consumption frequency and two questions to food intake habits considered characteristic of the MedDiet. Each answer to those questions was coded as 0 (when the MEDAS condition was not met) or 1 (when the MEDAS condition was met).

Score one was associated with the following answers: consuming: (i) four or more tablespoons of olive oil/day; (ii) two or more servings of vegetables/day; (iii) three or more pieces of fruit/day; (iv) less than one serving of red meat or sausages/day; (v) less than one serving of animal fat/day; (vi) less than one cup of sugar-sweetened beverages/day; (vii) seven or more servings of red wine/week; (viii) three or more servings of legumes/week; (ix) three or more servings of fish/week; (x) fewer than two commercial pastries/week; (xi) three or more servings of nuts/week; (xii) two or more servings/week of a dish with a traditional sauce of tomatoes, garlic, onion, or leeks sautéed in olive oil and in the last group of two answers, the following two were preferred: (xiii) olive oil prime source of fat for cooking and (xiv) white meat over red meat. If those conditions were not met, 0 points were recorded. The final score ranged from 0 to 14. Adherence to the MedDiet was assessed by using two scales (24), where the first scale was a continuous one and ranged from 0 to 14 (MEDAS), while the other scale was categorical, allowing the classification of the respondents into low (MEDAS <5), medium (MEDAS: 5–9) and high (MEDAS >9) adherence levels. The MEDAS score before the confinement was calculated as proposed by Rodríguez-Pérez et al. (24).

Diet quality was evaluated through comparison with the MEDAS, since Croatia as a Mediterranean country encourages Mediterranean dietary pattern as a referent, ever since the Ancel Key's Seven Countries Study (Croatia included) (26).

### Statistical Analyses

All collected answers were coded as qualitative or quantitative data. Based on the data type, different tests were applied in the analysis. First, the descriptive statistics was conducted for presentation of adherence level to the MedDiet (gender, age, place of residence, regions, children in care, education levels, and the body mass index). From the test, which allows evaluating differences in means or proportions by observed variables across the strata, we used Students' *t*-test (for continuous normal distributed data); Kruskal–Wallis test (for non-normal distributed data), and Chi-squared test (for categorical data). Box-plots were also used to evaluate further the distribution of the variable on adherence to the MedDiet by the aforementioned subgroups.

Logistic regression model was used to explore variable changes with the change in cooking pattern during the confinement (cooking more vs. cooking as usual or less, as reference). Crude regression model (URM) included only the absence or presence of increased cooking habits, while the bivariate-adjusted model included age, gender, and region (Model 1). To remove their influence on the cooking habit, the next bivariate-adjusted model (Model 2) included Model 1 observations and physical activity, education level, and residence. Odds ratios (ORs) with corresponding 95% confidence intervals (CIs) were estimated for all models (Crude, Model 1, and Model 2). The OR >1 indicates greater odds of association with the exposure and outcome or increased occurrence of the examined event. In the squared brackets, the 95% confidence interval is listed for the OR, and if it includes 1, the result is not statistically significant. Software in data analyses SPSS v. 17 and XLStat for Excel 2013 were used.

## Results

### Respondents' Sociodemographic Characteristics by the Level of Adherence to Mediterranean Diet During the COVID-19 Confinement

The study sample consisted of 4,281 Croatian adults who completed the questionnaire. The main sociodemographic characteristics of respondents by levels of adherence to the Mediterranean diet (low, medium, and high) during the COVID-19 confinement in Croatia are shown in Table 1.

**Table 1:** Outlines of questionnaire responses by the level of adherence to the Mediterranean diet, based on the Mediterranean diet adherence screener (MEDAS) of Croatian respondents during COVID-19 confinement.

**Respondents' characteristics**	**MEDAS groups**								***P*-value^**c**^**
	**Low**		**Medium**		**High**		**All**		
	**N_**L**_ = 1,315**	**%**	**N_**M**_ = 2,806**	**%**	**N_**H**_ = 160**	**%**	***N* = 4,281**	**%**	
Gender^a^									<0.001
Female	937	71.3	2,365	84.3	142	88.8	3,444	80.5	
Male	376	28.6	435	15.5	18	11.2	829	19.4	
Age									0.252
<20 years	61	4.6	124	4.4	1	0.6	186	4.3	
20–35 years	554	42.1	1,267	45.2	71	44.4	1,892	44.2	
36–50 years	510	38.8	1,049	37.4	63	39.4	1,622	37.9	
51–65 years	179	13.6	339	12.1	23	14.4	541	12.6	
>65 years	11	0.8	27	1.0	2	1.3	40	0.9	
Place of Residence									0.360
Alone	127	9.7	328	11.7	12	7.5	467	10.9	
Family home	1,098	83.5	2,298	81.9	136	85.0	3,532	82.5	
Shared flat	79	6.0	163	5,8	11	6.9	253	5.9	
Student's residence	11	0.8	17	0.6	1	0.6	29	0,7	
Region by Areas^b^									<0.001
Continental part	1,148	87.3	2,238	79,8	119	74.4	3,505	81.9	
Coastal part	167	12.7	568	20.2	41	25.6	776	18.1	
Children in Care									0.284
No	775	58.9	1,690	60.2	87	54.4	2,552	59.4	
Yes	540	41.1	1,116	39.8	73	45.6	1,729	40.4	
Educational Level									<0.001
University	661	50.3	1,597	56.9	106	66.3	2,364	55.2	
Postgraduate	247	18.8	536	19.1	33	20.6	816	19.1	
Professional	393	29.9	652	23.2	21	13.1	1,066	24.9	
Primary	14	1.1	21	0.7	0	0.0	35	0.8	
BMI									<0.001
Underweight	39	3.0	88	3.1	5	3.1	132	3.1	
Normal	726	55.2	1,901	67.7	113	70.6	2,740	64.0	
Overweight	387	29.4	609	21.7	36	22.5	1,032	24.1	
Obesity	163	12.4	208	7.4	6	3.8	377	8.8	

a *Numbers do not add up due to eight respondents who stated that by gender they belong to the category of “Other” (data not shown)*.

b *Official EU division areas for Croatia—NUTS 2 (27).*

c *Differences between the three Mediterranean diet adherence groups were evaluated by the Chi-squared test*.

Out of all the respondents, 80.5% were females. The majority resided in the continental part of Croatia (81.9%) in family homes (82.5%). A small number of respondents were in the youngest (4.3%) and the oldest age group (0.9%), while 44.2 and 37.9% were aged 20–35 and 35–50, respectively. The highest number of respondents (55.2%) was registered as having university level of education. Moreover, 59.6% did not have children in care and were self-assessed as normal category of body mass index (64.0%).

**Figure 1:**
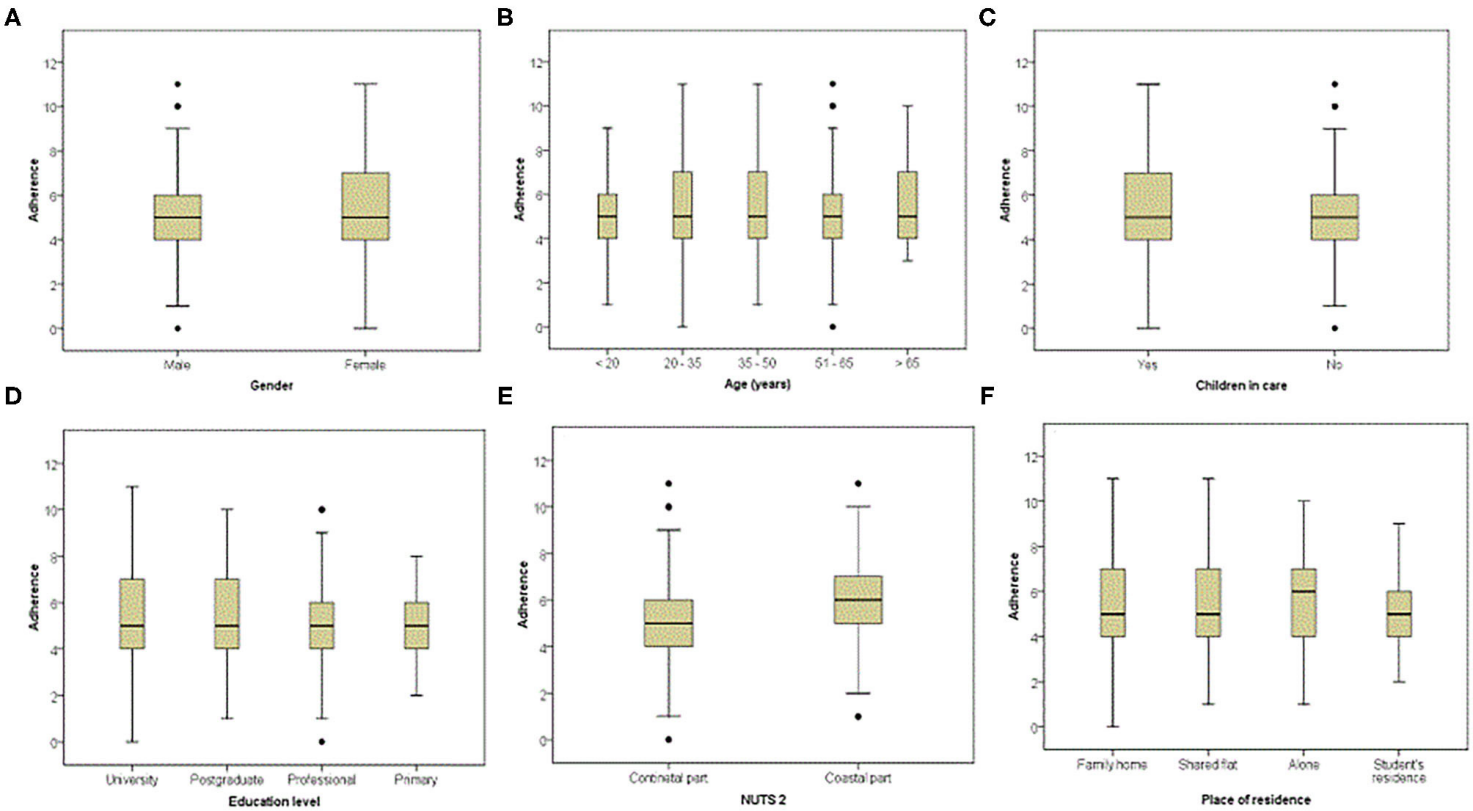
Adherence to the Mediterranean diet based on the Mediterranean diet adherence screener (MEDAS), during the COVID-19 confinement by subgroups, presented as box plots. The interquartile range (IQR) or box boundaries are the 25th percentile, Q1 (box closest to x-axis) and the 75th percentile, Q3 (farthest from x-axis). The line within the box presents the median, Q2 (50th percentile). Lines below or above the box indicate the minimum (Q1–1.5^*^IQR) or maximum (Q3 + 1.5^*^IQR), while the dots above and below those lines present outliers.

Respondents who had higher adherence to the MedDiet were mostly females (88.8%), aged between 20 and 50 years old, with the highest level of education (66.3%), and normal BMI (70.6%), who lived in family homes (85.0%) in the continental part of Croatia (74.4%) (Table 1). However, observing the subset data for region in box plot structure, higher values of the MEDAS were indicated for respondents residing in the coastal part of Croatia, as for those respondents who lived alone (Figure 1).

### Respondents' Dietary Behavior by the Level of Adherence to the Mediterranean Diet During the COVID-19 Confinement

Dietary behaviors related to the adherence to the MedDiet are described in Table 2. During the COVID-19 confinement, 20.3, 22.1, and 16.3% respondents met the MEDAS condition for olive oil, vegetable, and fruit consumption, respectively. The lowest group of olive oil consumption (0–1.9 tablespoons/day) was noted in 45.6% of respondents, while for vegetable and fruit consumption, most respondents had medium consumption (60.2 and 56.8%). Interestingly, a great number of respondents have met the MEDAS criterion for consumption of red meat (69.8%), as well as for consumption of animal fat (82.8%) and sugar-sweetened beverages (91.8%). The lowest degree of satisfaction was noted for MEDAS conditions of wine (3.0%), legumes (10.7%), and fish and seafood consumption (5.1%). Commercial pastry consumption criterion was met in 61.2% of the respondents, consumption of a dish with a traditional sauce of tomatoes, garlic, onion, or leeks sautéed in olive oil in 59.4%, while the MEDAS condition for nuts consumption was satisfied in 25.3% of the respondents. The majority (70.1%) of the respondents preferred white over red meat, while olive oil was not preferred in 52.7% of the respondents.

**Table 2:** Eating behavior by the level of adherence to the Mediterranean diet, based on the Mediterranean diet adherence screener (MEDAS) of Croatian respondents during COVID-19 confinement.

**Observed eating behavior**	**MEDAS groups**								***P*-value^**a**^**
	**Low**		**Medium**		**High**		**All**		
	**N_**L**_ = 1,315**	**%**	**N_**M**_ = 2,806**	**%**	**N_**H**_ = 160**	**%**	**N = 4,281**	**%**	
Olive oil consumed (tablespoons (13.5 g)/day)									<0.001
0–1.9	915	69.9	1,032	36.8	6	3.8	1,953	45.6	
2–3.9	330	25.1	1,093	39.0	37	23.1	1,460	34.1	
>4	70	5.3	681	24.3	117	73.1	868	20.3	
Vegetables consumed (servings (200 g)/day)									<0.001
0–0.9	370	28.1	384	13.7	4	2.5	758	17.7	
1–1.9	870	66.2	1,675	59.7	31	19.4	2,576	60.2	
>2	75	5.7	747	26.6	125	78.1	947	22.1	
Fruit consumed (pieces/day)									<0.001
0–0.9	515	39.2	620	22.1	17	10.6	1,152	26.9	
1–2.9	738	56.1	1,645	58.6	49	30.6	2,432	56.8	
>3	62	4.7	541	19.3	94	58.8	697	16.3	
Red meat consumed (servings (100–150 g)/day)									<0.001
0–0.9	553	42.1	2,286	81.5	151	94.4	2,990	69.8	
>1	762	57.9	520	18.5	9	5.6	1,291	30.2	
Butter, margarine or cream consumed (servings (12 g)/day)									<0.001
0–0.9	909	69.1	2,478	88.3	156	97.5	3,543	82.8	
>1	406	30.9	328	11.7	4	2.5	738	17.2	
Carbonated and/or sugary beverages consumed (times/day)									<0.001
0–0.9	1,094	83.2	2,678	95.4	160	100.0	3,932	91.8	
>1	221	16.8	128	4.6	0	0.0	349	8.2	
Wine consumed (cups (100 ml)/week)									<0.001
0–2.9	643	48.9	1,362	48.5	68	42.5	2,073	48.4	
3–6.9	96	7.3	223	7.9	13	8.1	332	7.8	
>7	22	1.7	85	3.0	20	12.5	127	3.0	
I never drink wine	554	42.1	1,136	40.5	59	36.9	1,749	40.9	
Legumes consumed (servings (150 g)/week)									<0.001
0–0.9	570	48.3	848	30.2	16	10.0	1,434	33.5	
1–2.9	721	54.8	1,612	57.4	58	36.3	2,391	55.9	
>3	24	1.8	346	12.3	86	53.8	456	10.7	
Fish-seafood consumed (servings (100–150 g for fish or 200 g for seafood)/week)									<0.001
0–0.9	791	60.2	1,219	43.4	32	20.0	2,042	47.7	
1–2.9	517	39.3	1,432	51.0	70	43.8	2,019	47.2	
>3	7	0.5	155	5.5	58	36.3	220	5.1	
Commercial pastries consumed (times/week)									<0.001
0–1.9	901	68.5	1,637	58.3	81	50.6	2,619	61.2	
>2	414	31.7	1,169	41.7	79	49.4	1,662	38.8	
Nuts consumed (servings (30 g)/week)									<0.001
0–0.9	733	55.7	1,020	36.4	24	15.0	1,777	41.5	
1–2.9	424	32.2	959	34.2	39	24.4	1,422	33.2	
>3	158	12.1	827	29.4	97	60.6	1,082	25.3	
Vegetables, pasta, rice cooked in olive oil (times/week)									<0.001
0–0.9	305	23.2	1,74	6.2	5	3.1	484	11.3	
1–1.9	622	47.3	626	22.3	4	2.5	1,252	29.2	
>2	388	29.5	2,006	71.5	151	94.4	2,545	59.4	
Preferred Olive Oil for Cooking									<0.001
No	1,100	83.7	1,145	40.8	12	7.5	2,257	52.7	
Yes	215	16.3	1,661	59.2	148	92.5	2,024	47.3	
Preferred white meat instead of red meat									<0.001
No	712	54.1	555	19.8	11	6.9	1,278	29.9	
Yes	603	45.9	2,251	80.2	149	93.1	3,003	70.1	

a *Differences between the three Mediterranean diet adherence groups were evaluated by the Chi-squared test*.

As expected, the majority of respondents within the high MEDAS group had high consumption of olive oil (73.1%), vegetables (78.1%), and fruit (58.8%), as well as high weekly consumption of legumes (53.8%), and medium fish and seafood consumption (43.8%). Low red meat, animal fat, and sugar-sweetened beverage consumption was recorded in 94.4, 97.5, and 100.0% of respondents, respectively. Nut consumption and meals with tomatoes, garlic, and onion were noted in the highest rate of consumption in the high MEDAS group. Moreover, 92.5% of high MEDAS respondents preferred the use of olive oil in cooking, and 93.1% preferred white over red meat (Table 2).

Although significant differences across the three MEDAS groups were found for all 14 MEDAS conditions (Table 2), low consumption of animal fats, sugar-sweetened beverages, and commercial pastries were noted in all groups. Furthermore, wine consumption MEDAS criterion was indicated as the least satisfied one in all the MEDAS groups. However, respondents in the high MEDAS group showed the highest rate of satisfying this criterion (12.5%).

### Changes in Dietary Behavior and Styles by the Level of Adherence to the Mediterranean Diet During the COVID-19 Confinement

Changes in eating behavior and lifestyles by the level of adherence to the MedDiet during COVID-19 confinement in Croatia are described in Tables 3, 4. The majority of respondents maintained their dietary behavior as before COVID-19 confinement (Table 3). In all MedDiet adherence groups, a lower intake of carbonated and/or sugar-sweetened drinks (23.9–27.5% of respondents) as well as alcoholic beverage intake (26.3–27.5%) was noted. Also, a higher intake of vegetables, fruits, and legumes during the confinement was observed in all MEDAS groups. Respondents with a high adherence to MedDiet showed the highest rate of positive changes in these three groups of food (Table 3).

**Table 3:** Changes in eating behavior by the level of adherence to the Mediterranean diet, based on the Mediterranean diet adherence screener (MEDAS) of Croatian respondents during the COVID-19 confinement.

	**MEDAS groups**								***P*-value^**a**^**
	**Low**		**Medium**		**High**		**All**		
	**N_**L**_ = 1,315**	**%**	**N_**M**_ = 2,806**	**%**	**N_**H**_ = 160**	**%**	**N = 4,281**	**%**	
Olive oil consumed (tablespoons/day)									<0.001
As usual	1,087	82.7	2,259	80.5	120	81.3	3,476	81.2	
Lower	118	9.0	152	5.4	3	1.9	273	6.4	
Higher	110	8.4	395	14.1	27	16.9	532	12.4	
Vegetable consumed (servings/day)									<0.001
As usual	950	72.2	1,931	68.8	109	68.1	2,990	69.8	
Lower	15	11.5	228	8.1	6	3.8	385	9.0	
Higher	214	16.3	647	23.1	45	28.1	906	21.2	
Fruit consumed (servings/day)									0.001
As usual	917	69.7	1,920	68.4	103	64.4	2,940	68.7	
Lower	153	11.6	248	8.8	13	8.1	414	9.7	
Higher	245	18.6	638	22.7	44	27.5	927	21.7	
Red meat consumed (servings/day)									<0.001
As usual	934	71.0	1,917	68.3	101	63.1	2,952	69.0	
Lower	187	14.2	695	24.8	54	33.8	936	21.9	
Higher	194	14.8	194	6.9	5	3.1	363	9.2	
Carbonated and/or sugary beverages consumed (cups/day)									<0.001
As usual	900	69.4	1,937	69.0	113	70.6	2,950	69.9	
Lower	314	23.9	738	26.3	44	27.5	1,096	25.6	
Higher	101	7.7	131	4.7	3	1.9	235	5.5	
Legumes consumed (servings/week)									<0.001
As usual	1,103	83.9	2,332	83.1	119	74.4	3,554	83.0	
Lower	90	6.8	167	6.0	4	2.5	261	6.1	
Higher	122	9.3	307	10.9	37	23.1	466	10.9	
Fish-seafood consumed (servings/week)									<0.001
As usual	1,018	77.4	1,974	70.3	102	63.8	3,094	72.3	
Lower	189	14.4	443	15.8	20	12.5	652	15.2	
Higher	108	8.2	389	13.9	38	23.8	535	12.5	
Commercial pastries consumed (units/week)									0.001
As usual	852	64.8	1,592	56.7	95	59.4	2,539	59.3	
Lower	197	15.0	593	21.1	30	18.8	820	19.2	
Higher	266	20.2	621	22.1	35	21.9	922	21.5	
Homemade pastries consumed (units/week)									0.147
As usual	744	56.6	1,469	52.4	89	55.6	2,302	53.8	
Lower	154	11.7	371	13.2	19	11.9	544	12.7	
Higher	417	31.7	966	34.4	52	32.5	1,435	33.5	
Alcoholic beverage consumed									0.760
As usual	832	63.3	1,729	61.6	102	63.7	2,663	62.2	
Lower	361	27.5	784	27,9	42	26.3	1,187	27.7	
Higher	122	9.3	293	10.4	16	10.0	431	10.1	

a *Differences between the three Mediterranean diet adherence groups were evaluated by the Chi-squared test*.

**Table 4:** Cooking preparation practice, changes in eating behavior and life styles by the level of adherence to the Mediterranean diet, based on the Mediterranean diet adherence screener (MEDAS) of Croatian respondents during the COVID-19 confinement.

	**MEDAS groups**								***P*-value^**a**^**
	**Low**		**Medium**		**High**		**All**		
	**N_**L**_ = 1,315**	**%**	**N_**M**_ = 2,806**	**%**	**N_**H**_ = 160**	**%**	**N = 4,281**	**%**	
Difficult to find certain types of food									0.053
No	1,084	82.4	2,215	78.9	122	76.3	3,421	79.9	
Yes	231	17.6	591	21.1	38	23.8	860	20.1	
Type of cooking									<0.001
Fried	132	10.0	108	3.8	3	1.9	243	5.7	
Griddle	26	2.0	31	1.1	3	1.9	60	1.4	
Microwave	7	0.5	5	0.2	0	0.0	12	0.3	
Oven	242	18.4	354	12.6	18	11.3	614	14.3	
Stew	908	69.0	2,308	82.3	136	85.0	3,352	78.3	
Cooking more often									<0.001
As usual	639	48.6	1,172	41.8	72	45.0	1,883	44.0	
Less	35	2.7	61	2.2	0	0.0	96	2.2	
More	641	48.7	1,573	56.1	88	55.0	2,302	53.8	
Fried foods consumed									<0.001
As usual	937	71.3	1,913	68.2	116	72.5	2,966	69.3	
Lower	248	18.9	739	26.3	44	27.5	1,031	24.1	
Higher	130	9.9	154	5.5	0	0.0	284	6.6	
Frequency of fried food consumed (times/week)									<0.001
1–3	836	63.6	1,380	49.2	58	36.3	2,274	53.1	
4–6	87	6.6	80	2.9	2	1.3	169	3.9	
>7	5	0.4	8	0.3	0	0.0	13	0.2	
<1	341	25.9	1,138	40.6	79	49.4	1,558	36.4	
Never	46	3.5	200	7.1	21	13.1	267	6.2	
Type of oil used for frying									<0.001
Olive oil	134	10.2	764	27.2	80	50.0	978	22.8	
Other	166	12.6	384	13.7	19	11.9	569	13.3	
Sunflower oil	1,015	77.2	1,658	59.1	61	38.1	2,734	63.9	
Frequency of snacking									0.017
As usual	748	56.9	1,504	53.6	84	52.5	2,336	54.5	
Lower	148	11.3	316	11.3	29	18.1	493	11.5	
Higher	419	31.9	986	35.1	47	29.4	1,452	33.9	
Frequency of fast food consumed									0.001
As usual	575	43.7	1,201	42.8	85	53.1	1,861	43.5	
Lower	694	52.8	1,553	55.3	72	45.0	2,319	54.2	
Higher	46	3.5	52	1.9	3	1.9	101	2.4	
Ate more									0.066
No	736	56.0	1,593	56.8	105	65.6	2,434	56.9	
Yes	579	44.0	1,213	43.2	55	34.4	1,847	43.1	
Modification of physical activity									<0.001
No physical activity	142	10.8	157	5.6	5	3.1	304	7.1	
It has decreased	483	36.7	1,047	37.3	51	31.9	1,581	36.9	
It has increased	329	25.0	840	29.9	61	38.1	1,230	28.7	
It remains as usual	361	27.5	762	27.2	43	26.9	1,166	27.2	
Weight gain									0.013
I do not know	308	23.4	616	22.0	23	14.4	947	22.1	
No	716	54.4	1,643	58.6	104	65.0	2,463	57.5	
Yes	291	22.1	547	19.5	33	20.6	871	20.3	
Meals consumed (number/day)^b^									<0.001
1	572	43.5	1,153	41.1	67	41.9	1,792	41.9	
2	298	22.7	596	21.2	13	8.1	907	21.2	
3	209	15.9	422	15.0	30	18.8	661	15.4	
None	236	17.9	635	22.6	50	31.3	921	21.5	

a *Differences between the three Mediterranean diet adherence groups were evaluated by the Chi-squared test*.

b *Referred as the usual number of meals out of home before the confinement*.

However, differences regarding changes in eating behavior were also detected among all the MEDAS groups. Medium and high adherence to MedDiet groups showed similar changes regarding higher olive oil and lower red meat consumption during the confinement. Those changes were more prominent in the high MEDAS group than in the medium one. In contrast, the group with low adherence to MedDiet showed opposite changes, with an interesting result where 14.8% of the participants in this group showed higher red meat consumption during confinement. However, fish and seafood consumption did not show the same trend of change as the other dietary intake groups. Higher consumption of a previously mentioned food group during the confinement was noted only for the group with high adherence to the MedDiet, while respondents in low and medium MEDAS groups described their intake of fish and seafoods as lower than before COVID-19 confinement.

Overall, most respondents employed stewing as the main type of cooking technique during the COVID-19 confinement (78.3%). Fried food consumption remained as usual in 69.3% of respondents, which means that 53.1% of them consumed fried food one to three times a week, while 36.4% ate fried food less than once a week (Table 4). As expected, the respondents in high adherence to the MedDiet group showed lower tendency to fried food consumption, namely, 49.4% of them consumed this type of food less than once a week. Regarding the type of oil used for frying, the majority of respondents (63.9%) used sunflower oil, while the highest preference for the use of olive oil (50.0%) was found in respondents with high adherence to MedDiet. Out of all respondents, 41.9% of them consumed one meal per day (out of home), while 21.5% did not consume any meal out of home before the confinement.

All groups agreed that they did not have trouble finding certain types of food, while 56.9% of the respondents stated that their amount of food eaten was the same as before the confinement. Also, 57.5% did not perceive any change in body mass. Interestingly, the highest rate of gaining weight (22.1%) was noted in the low MEDAS group (Table 4). Furthermore, modification in physical activity was observed in 36.9% of the respondents who decreased, while 28.7% of them increased their physical activity. Respondents in the high MEDAS group dominantly increased their physical activity (38.1%) level during COVID-19 confinement, while the other two groups showed higher rate of decrease than increase in physical activity.

Moreover, 29.4–35.1% of the respondents, depending on the MEDAS group, had higher frequency of snacking. In 54.2% of the respondents, a lower intake of fast food was found during the confinement, while 53.8% of the overall respondents cooked more often (Table 4) and based on the presented share of students and young working people (aged 20–35 years) and those who live in family homes, it seems that the cooking was a shared responsibility in the household. Still, a difference in behavior change among different MEDAS groups was noted (*p* < 0.001), where the respondents in the low MEDAS group were almost equally divided in the cooking as usual group (48.6%) and the cooking more group (48.7%).

**Figure 2:**
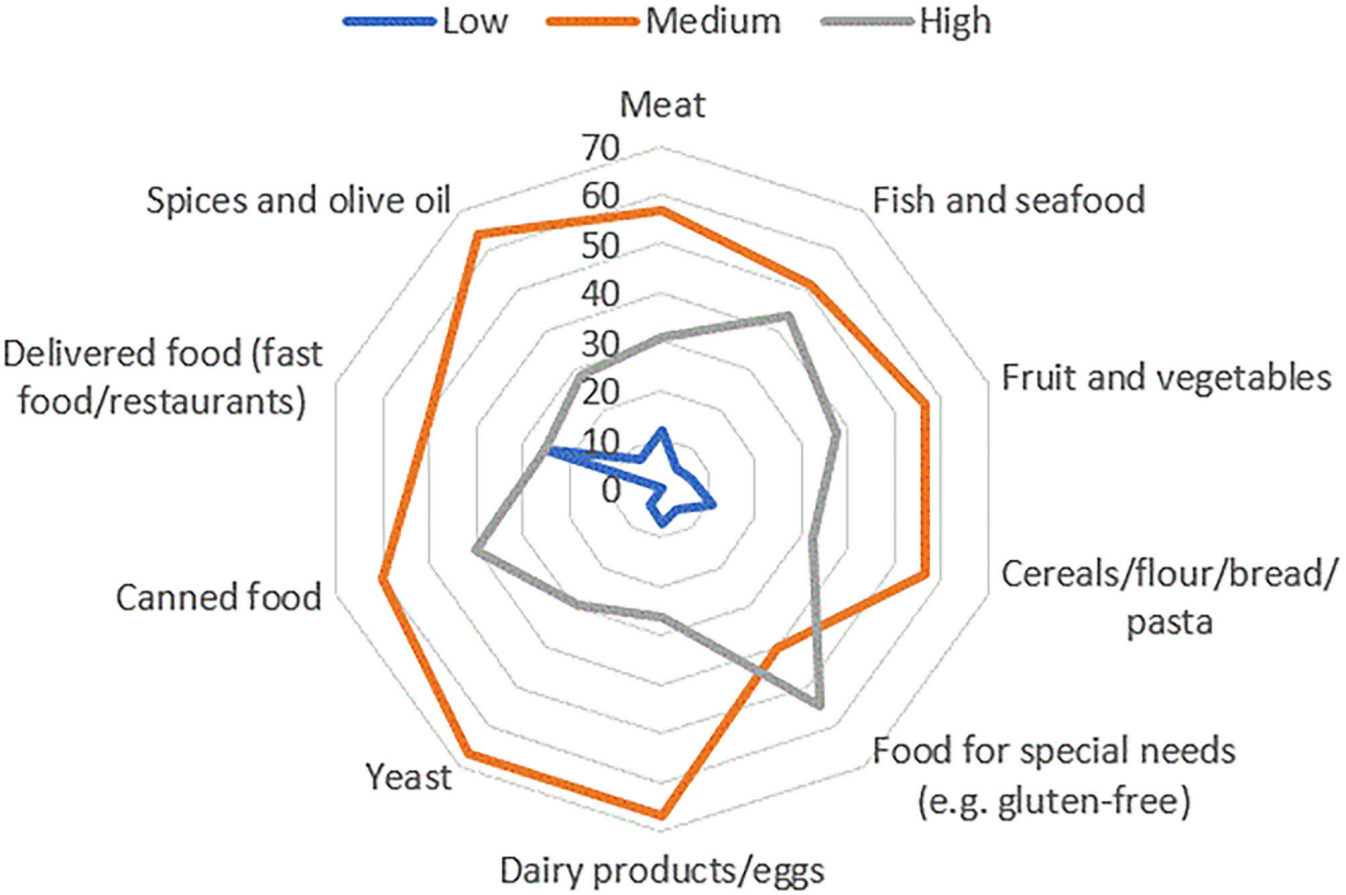
Graphical representation of types of food, which respondents had difficulty purchasing during COVID-19 confinement in Croatia. Respondents' answers were divided in accordance with the level of adherence to the Mediterranean diet (low, medium, and high).

While the majority of respondents had no difficulty finding certain types of food during the confinement, 20.1% of the respondents described which type of food was troubling to purchase (Table 4 and Figure 2). The respondents who scored medium MEDAS were the most eager to describe problematic types of food. They had difficulty finding a wide range of different foods, especially yeast, dairy products, and eggs, as well as spices and olive oil (Figure 2). The group with high adherence to MedDiet found food for special needs the most difficult to purchase during the confinement, mainly organic, vegan, and gluten-free food. They also had problems finding fish and seafood, as well as canned food. Interestingly, the respondents in the low MEDAS group were the most worried about food delivery, as well as difficulty to consume fast food and food from restaurants (Figure 2).

### Adherence to Mediterranean Diet and Changes in Dietary Behavior During the COVID-19 Confinement Regarding Body Mass Index of Respondents

**Figure 3:**
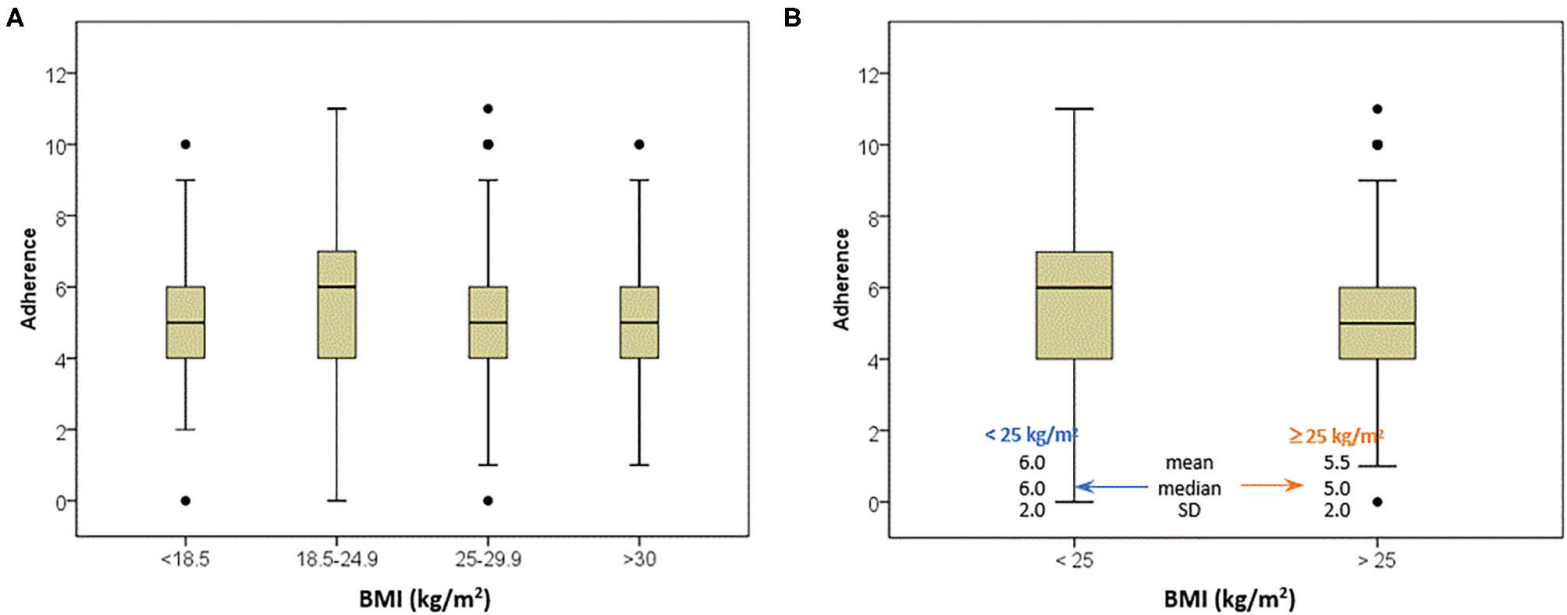
Box plots of the body mass index of respondents according to adherence with the Mediterranean diet pattern.

Higher median values of MEDAS score were noted for respondents with BMI values between 18.5 and 24.9 kg/m^2^. An interesting result, indicating a higher median value of MEDAS score for respondents with a BMI <25 kg/m^2^ (6.0) than those with a BMI value above 25 kg/m^2^ (5.0) was registered (Figure 3). Statistically significant differences between those two groups were noticed (*p* < 0.001) regarding the frequency of consuming fried food and increase in snacking during the COVID-19 confinement, where respondents with higher BMI values showed higher intake of fried food, but lower frequency of snacking during the confinement in comparison with the number of snacks before the confinement. Even though the difference between the two groups was not registered for eating more during the confinement (*p* = 0.401), a higher probability of weight gain was observed for respondents with BMI above 25 kg/m^2^. Also, respondents with a BMI <25 kg/m^2^ were more prone to increasing their physical activity during the confinement than respondents with higher BMI values (Supplementary Table 1).

The models presenting the OR are presented in Supplementary Table 2. Odds ratio is defined as the ratio of changes in dietary behavior of respondents with BMI above 25 kg/m^2^ to the changes in dietary behavior of respondents with a BMI below 25 kg/m^2^, as a measure of association between the “exposure” and the outcome. Here, the outcomes were responses of respondents with a BMI >25 kg/m^2^, while the changes in dietary behavior were used as the exposures. The used models confirm previous results that the respondents with higher BMI had 37% higher likelihood to decrease their frequency of snacking during the confinement. While models do not indicate higher intake of fried food for the respondents with BMI above 25 kg/m^2^, a lower consumption of sugar-sweetened beverages, and commercial and homemade pastries was noted for those respondents. In spite of the results showing beneficial changes, models, in accordance with previous results, show that the odds are 1.76 higher for weight gained by respondents with higher BMI values (Supplementary Table 2).

### The Change in Cooking Frequency During the COVID-19 Confinement

Considering gender (Supplementary Table 3), 2.1% of female respondents cooked less, while 56.1% of them increased the cooking frequency, as well as 44.3% of the male respondents, respectively. Respondents in all residence places increased the cooking frequency during the confinement as well as those who had children in care (57.0%), had higher education levels (university degree: 59.2%; postgraduate level: 52.9%), aged 20–50 years, and with medium or high MEDAS (56.1 and 55.0%, respectively).

The models presenting the OR are presented in Supplementary Table 5. In our study, the outcome was the cooking practice, while the factors which were scored in the MEDAS were used as the exposures. We can conclude that greater consumption of wine was noted (OR = 1.79 for 3–6.9 cups/week) for the respondents who cooked more during the confinement, while preferring olive oil for cooking and white meat consumption did not show a correlation within the group cooking more (Supplementary Table 5). The results of multivariate analysis did not show significant association of consumption of MedDiet-related food groups in the group that cooked more during the confinement, so the cross table observing 14 parameters for the MEDAS evaluation by the change in cooking frequency was conducted (Supplementary Table 4). For those respondents who increased the cooking frequency—significant changes are in the MEDAS i, vii, x, xi, xii, and xiii. Respondents who decreased their cooking frequency drank three or more cups of wine per week, but also 45.8% of them never drank wine. The respondents who cooked more during the confinement had higher preference for the use of olive oil as main source of added fat (51.2%) in comparison with the respondents who cooked as usual (43.1%) or less (35.4%). However, for the preference of white over red meat, no statistically significant changes were noted (*p* = 0.263). Those who cooked more and as usual exhibited similar results (71.0 vs. 69.4%, respectively), while the respondents who cooked less showed lower preference for white meat (Supplementary Table 4). However, the respondents with increased cooking frequency during the confinement also had higher olive oil and nut intake as well as higher weekly consumption of vegetables, pasta, or rice cooked in olive oil (Supplementary Table 4), which was not confirmed by multivariate analysis (Supplementary Table 5).

To elucidate the association between changes in food frequency consumption and cooking frequency during the COVID-19 confinement, models regarding the cooking practice as outcome and the changes in dietary behavior as the exposures were made (Table 5). Following the results from the previously presented models where those respondents who cooked more had higher wine intake, these models also show higher alcoholic beverage consumption. Furthermore, the respondents who increased their cooking frequency showed 2.24 higher odds of higher vegetable intake during the confinement. Along with the increased vegetable consumption, respondents who cooked more consumed more legumes (OR = 1.38), fish and seafood (OR = 1.33), as well as homemade pastries. However, they also exhibited higher frequency of snacking and consumption of fried food, but lower intake of fast food (Table 5).

**Table 5:** Associations between the change in cooking practice and the change in dietary behavior of Croatian respondents during the COVID-19 confinement.

	**As usual or less**		**Cooking more**		**All**		***P*-value^**a**^**	**Crude** ^****b****^		**Model 1** ^****c****^		**Model 2** ^****d****^	
	***N* = 1,979**	**%**	***N* = 2,302**	**%**	***N* = 4,281**	**%**		**OR**	**95%CI**	**OR**	**95%CI**	**OR**	**95%CI**
Vegetable consumed							<0.001						
Higher	239	12.0	667	29.0	906	21.2		**2.24**	**[1.86;2.71]**	**2.17**	**[1.79;2.63]**	**2.18**	**[1.79;2.65]**
Lower	145	7.3	240	10.4	385	9.0		**1.37**	**[1.05;1.79]**	**1.41**	**[1.08;1.85]**	**1.35**	**[1.02;1.77]**
As usual	1,595	79.9	1,395	60.6	2,990	69.8		Ref.		Ref.		Ref.	
Fruit consumed							0.512						
Higher	315	15.9	612	26.6	927	21.7		1.08	[0.90;1.29]	1.14	[0.95;1.37]	1.14	[0.95;1.37]
Lower	174	8.8	240	10.4	414	9.7		0.92	[0.71;1.18]	0.87	[0.67;1.12]	0.90	[0.69;1.17]
As usual	1,490	75.3	1,450	63.0	2,940	68.7		Ref.		Ref.		Ref.	
Red meat consumed							0.131						
Higher	121	6.1	272	11.8	393	9.2		1.22	[0.94;1.58]	1.27	[0.98;1.66]	1.27	[0.97;1.66]
Lower	358	18.1	578	25.1	936	21.9		1.16	[0.97;1.39]	1.18	[0.98;1.42]	1.14	[0.95;1.38]
As usual	1,500	75.8	1,452	63.1	2,952	69.0		Ref.		Ref.		Ref.	
Carbonated and/or sugary beverages consumed							0.200						
Higher	79	4.0	156	6.8	235	5.5		0.98	[0.71;1.35]	1.02	[0.74;1.40]	1.09	[0.78;1.51]
Lower	456	23.0	640	27.8	1.096	25.6		0.85	[0.72;1.02]	0.86	[0.72;1.02]	0.90	[0.75;1.07]
As usual	1,444	73.0	1,506	65.4	2.950	68.9		Ref.		Ref.		Ref.	
Legumes consumed							0.025						
Higher	130	6.6	336	14.6	466	10.9		**1.38**	**[1.09;1.76]**	**1.37**	**[1.08;1.74]**	**1.32**	**[1.03;1.68]**
Lower	99	5.0	162	7.0	261	6.1		0.98	[0.73;1.32]	0.98	[0.72;1.33]	1.02	[0.75;1.38]
As usual	1,750	88.4	1,804	78.4	3,554	83.0		Ref.		Ref.		Ref.	
Fish-seafood consumed							0.035						
Higher	168	8.5	367	15.9	535	12.5		**1.33**	**[1.07;1.66]**	**1.34**	**[1.08;1.68]**	1.26	[1.00;1.58]
Lower	260	13.1	392	17.0	652	15.2		1.09	[0.89;1.33]	1.07	[0.87;1.31]	1.00	[0.81;1.24]
As usual	1,551	78.4	1,543	67.0	3,094	72.3		Ref.		Ref.		Ref.	
Commercial pastries consumed							0.077						
Higher	322	16.3	600	26.1	922	21.5		0.93	[0.76;1.13]	0.91	[0.75;1.11]	0.88	[0.72;1.07]
Lower	313	15.8	507	22.0	820	19.2		1.23	[0.99;1.51]	1.23	[0.99;1.52]	1.19	[0.96;1.48]
As usual	1,344	67.9	1,195	51.9	2,539	59.3		Ref.		Ref.		Ref.	
Homemade pastries consumed							<0.001						
Higher	435	22.0	1,000	43.4	1,435	33.5		**2.01**	**[1.70;2.36]**	**1.98**	**[1.68;2.34]**	**2.09**	**[1.76;2.48]**
Lower	251	12.7	293	12.7	544	12.7		0.89	[0.70;1.13]	0.87	[0.68;1.11]	0.86	[0.67;1.10]
As usual	1,293	65.3	1,009	43.8	2,302	53.8		Ref.		Ref.		Ref.	
Alcoholic beverage consumed							<0.001						
Higher	119	6.0	312	13.6	431	10.1		**1.93**	**[1.51;2.46]**	**1.75**	**[1.37;2.25]**	**1.58**	**[1.23;2.03]**
Lower	508	25.7	679	29.5	1,187	27.7		0.86	[0.73;1.01]	0.90	[0.76;1.06]	0.89	[0.75;1.05]
As usual	1,352	68.3	1,311	57.0	2,663	62.2		Ref.		Ref.		Ref.	
Fried foods consumed							<0.001						
Higher	66	3.3	218	9.5	284	6.6		**2.37**	**[1.73;3.26]**	**2.52**	**[1.82;3.48]**	**2.73**	**[1.96;3.80]**
Lower	340	17.2	691	30.0	1,031	24.1		**1.62**	**[1.36;1.93]**	**1.64**	**[1.38;1.96]**	**1.63**	**[1.36;1.95]**
As usual	1,573	79.5	1,393	60.5	2,966	69.3		Ref.		Ref.		Ref.	
Frequency of snacking							<0.001						
Higher	437	22.1	1,015	44.1	1,452	33.9		**1.97**	**[1.67;2.31]**	**2.00**	**[1.69;2.36]**	**1.95**	**[1.65;2.31]**
Lower	235	11.9	258	11.2	493	11.5		0.95	[0.77;1.18]	0.96	[0.77;1.19]	0.92	[0.74;1.16]
As usual	1,307	66.0	1,029	44.7	2,336	54.6		Ref.		Ref.		Ref.	
Frequency of fast-food consumed							<0.001						
Higher	36	1.8	65	2.8	101	2.4		1.21	[0.75;1.96]	1.25	[0.77;2.04]	1.18	[0.72;1.93]
Lower	870	44.0	1,449	63.0	2,319	54.2		**1.68**	**[1.44;1.94]**	**1.68**	**[1.44;1.95]**	**1.74**	**[1.49;2.03]**
As usual	1,073	54.2	788	34.2	1,861	43.5		Ref.		Ref.		Ref.	
Weight gain							0.885						
Yes	322	16.3	549	23.9	871	20.4		1.05	[0.86;1.30]	1.07	[0.87;1.32]	1.07	[0.86;1.33]
No	1,238	62.6	1,225	53.2	2,463	57.5		1.02	[0.86;1.20]	1.02	[0.86;1.21]	1.00	[0.84;1.19]
I do not know	419	21.2	528	22.9	947	22.1		Ref.		Ref.		Ref.	

a *Differences between the groups were evaluated by the Chi-squared test*.

b *Crude model: unadjusted for any variable*.

c *Model 1: multivariate adjusted model for gender (women, men, and other), age groups (<20, 20–35 years, 36–50 years, 51–65 years, and >65 years), and regions (Continental and Coastal)*.

d *Model 2: multivariate adjusted model for gender, age groups, regions, residence (alone, family home, shared flat, and student's residence), education level (university, postgraduate, professional, and primary), and physical activity (higher, lower, similar, never). Odds ratios (ORs) and corresponding 95% confidence intervals were estimated for all models. In addition, statistically significant ORs are highlighted in bold*.

## Discussion

This research is aimed at investigating the effect of the COVID-19 confinement on dietary behaviors of adult population in Croatia. During the confinement, the major part of Croatian respondents presented medium adherence to the Mediterranean diet (5.85 ± 2.04). Our findings suggest that the respondents who increased their cooking frequency during the confinement also exhibited beneficial dietary behavior changes toward greater adherence to the MedDiet. Those respondents showed higher preference to the use of olive oil for cooking, 2.24 higher odds of increased vegetable consumption, as well as increased legumes and fish intake during the confinement.

Consumption of home-cooked meals on a regular basis was associated with an overall healthier diet (28), as well as with higher adherence to the MedDiet (29). The MedDiet may have a beneficial effect against viral infections and is therefore the advised eating approach during confinement (1, 18, 19). Adherence to this healthy and balanced nutritional pattern is important for achieving optimal function of the immune system and coping with stress and anxiety caused by an abnormal and disturbing situation worldwide. Owing to high fruit and vegetable intake, the MedDiet is abundant in antioxidants, especially vitamin E, vitamin C, and carotene, which play a crucial role in supporting the immune system. Also, vitamin D deficiency may cause a greater tendency for respiratory illnesses; therefore, the important role of this vitamin is emphasized. Due to the COVID-19 confinement policies, sun exposure was limited, which indicates a greater need for the intake of vitamin D from food and/or supplements (1, 18). Furthermore, adequate intake of omega-3 fatty acids supports the immune system, due to anti-inflammatory properties of their metabolites (12). Besides previously mentioned micronutrients, zinc is a trace element that is known for its role in maintaining the immune system (18). Te Velthuis et al. (30) have reported that increasing the intracellular level of zinc impairs the replication of RNA viruses, such as SARS-coronavirus in cell culture. All previously described nutrients are abundant in the MedDiet, so adherence to this healthy diet could be of great importance during the COVID-19 confinement.

Our study showed medium adherence to the MedDiet during the COVID-19 confinement. To be precise, the mean of the MEDAS score was 5.85 ± 2.04, and the mean of the MEDAS score before the confinement was 5.02 ± 1.97, which is significantly lower than the MEDAS score noted in Spain (24), but similar to that of the Danish population (31). This result might be due to the fact that the majority of respondents resided in the continental part of Croatia, whereas only 18.1% of the overall respondents were from the coastal part where higher adherence to the MedDiet is more common. Relative results suggest that the respondents in the coastal part of Croatia had higher MEDAS values than the respondents residing in the continental part.

High adherence to the MedDiet was noted in only 160 respondents (3.7% of all participants), while females showed higher adherence than male respondents, as seen in the Danish population (31). Similar to our results, female medical school students were associated with better adherence to the MedDiet (32, 33). Moreover, Papadaki et al. (34) reported a higher adherence in employed females. The same result was noted in Croatia, where employed females showed a higher MEDAS score than male participants (35). Furthermore, the importance of mothers in determining a better family diet quality has been reported by Schnettler et al. (36). As for female respondents, those respondents who lived in family homes also presented higher adherence to the MedDiet. A similar result was also noted in the Spanish population (24).

Considering the age of the respondents, those who were 20–50 years old presented the highest adherence to the MedDiet, which differs from the Spanish and Danish populations during the COVID-19 confinement, where respondents aged 51 years and older showed higher adherence to the MedDiet in comparison with the youngest ones (24, 31). The positive change in diet quality with increasing age was also suggested by Thiele et al. (37). Moreover, expected correlation between higher educational level and higher adherence to the MedDiet was found, as reported by Rodríguez-Pérez et al. (24).

While the majority of respondents rated their eating behavior as being the same as before the COVID-19 confinement, changes were noted in the remaining respondents. Lower consumption was noted for sugar-sweetened/carbonated beverages and alcohol. This result might be due to movement restriction, resulting in a reduction of time spent in local coffee shops and grocery stores. Moreover, Gray-Phillip et al. (38) reported that the usual on-premise (e.g., night bars) alcohol consumption was between midnight and 2 am, while for take-away alcohol (bought in stores), common purchase was made from 8 to 10 pm. On-premise consumption was consequently reduced because night clubs were not allowed to open during the confinement. Furthermore, grocery stores were closed by 5 pm, which may also result in less frequent purchase of take-away alcohol beverages. These findings were a probable reason for documented decrease in alcohol intake on an international level (39).

Respondents with medium and high adherence to the MedDiet also described higher olive oil and lower red meat consumption. These two MEDAS groups cover 69.3% of the overall respondents, so these results might suggest that the Croatian population increased their adherence to the MedDiet, as seen in the Spanish population (24).

Furthermore, the majority of the respondents consumed fried food during the COVID-19 confinement the same as before, that is, this type of food was consumed one to three times a week. As for the Croatian population, moderate intake of fried food was observed by Rodríguez-Pérez et al. (24). However, in contrast to the Spanish population, the Croatian population showed higher preference to frying in sunflower than in olive oil. The correlation between fried food consumption and overall diet quality is not well-addressed. Grosso et al. (40) documented that lower adherence to the MedDiet was associated with higher intake of sweets, sweetened beverages, fast food, and fried food, as well as with lower fruit, vegetable, pasta, cheese, and fish consumption in Italian adolescents. Furthermore, the correlation between fried food consumption and developing chronic diseases is inconsistent. The EPIC cohort study in Spain (41) showed that the consumption of fried food was not correlated with chronic diseases. Inconsistence in results regarding previously mentioned association was also stated by Gadiraju et al. (42). Our results suggest that 5.7% of the respondents reported frying as a type of cooking. This may suggest that they occasionally ordered food, which may be associated with higher intake of saturated fats and sweets, as well as lower consumption of nutrient-dense food such as fruits and vegetables (43, 44).

It is important to point out that confinement induces negative feelings such as stress and anxiety. Those feelings are usually accompanied by eating more of energy-dense food, especially in respondents who are overweight and obese (1). Sidor and Rzymski (45) have reported that eating and snacking more during the COVID-19 confinement were behaviors most frequently noticed in obese respondents. Apart from eating and snacking more, those respondents showed a tendency for higher intake of salty foods, meat, and dairy, while they consumed vegetables, fruits, and legumes less frequently. In overall responses, a higher frequency of snacking was noted (29.4–35.1% depending on MEDAS group), while snacking more during the confinement was also reported in the Danish and Lithuanian population (31, 46). Interestingly, respondents with BMI below 25 kg/m^2^ were more prone to increase the number of snacks during the confinement, which is not in accordance with the results presented by Sidor and Rzymski (45). This result may be due to boredom caused by staying at home. Along with higher frequency of snacking, respondents with BMI <25 kg/m^2^ were inclined to increase their physical activity, which may enable maintenance of energy balance. Out of all the respondents, 36.9% decreased their physical activity, as a consequence of the confinement, which is also reported by Ammar et al. (39). However, a recent study conducted by Lesser and Nienhuis (47) showed that modulation in physical activity during the COVID-19 confinement depends on the usual physical activity of respondents, whether they are usually active or inactive. In that study, active respondents had a higher level of physical activity, while inactive respondents described a lower level of physical activity during the confinement. Furthermore, the majority of respondents did not change the amount of eaten food, nor was there a change in body mass during the confinement perceived. Although the amount of consumed food was the same as before the confinement, the respondents with BMI above 25 kg/m^2^, as well as the respondents with lower adherence to the MedDiet, noticed an increase in body mass more frequently than the respondents with lower BMI values and higher adherence to the MedDiet. Although the results show beneficial changes in dietary behavior (lower snacking frequency, lower sugar-sweetened beverages, commercial and homemade pastry consumption) in respondents with higher BMI values, the odds are 1.76 higher for weight gain in those respondents.

Another expected result was that the respondents who had BMI values below 25 kg/m^2^ presented higher adherence to the MedDiet in comparison with the respondents with higher BMI values. It has been reported that high adherence to the MedDiet shows inverse association with BMI and obesity (48), as well as a beneficial role of the MedDiet in the prevention of weight gain, abdominal obesity (49), and protection from onset of chronic inflammation, type 2 diabetes, and metabolic syndrome (50).

Out of all the respondents who had difficulty finding certain types of food during the confinement, the respondents with medium and high adherence to the MedDiet found fruit and vegetables, as well as fish and seafood difficult to purchase. This might be explained by the usual purchase of these groceries on the market from local manufacturers, which were not allowed to work during the confinement. Moreover, along with markets being shut down, the Google Trends tool shows increased search for delivery of groceries by local manufacturers.

In order to limit the dissemination of viral infection, the recommendation was to stay home. This general shutdown policy resulted in several changes in lifestyle behavior. While the majority of respondents maintained almost all their behaviors the same as before the COVID-19 confinement, almost 54% of all the respondents stated that they cooked more frequently during the confinement, while their intake of fast food was lower. Generally speaking, there are some determinants involved in cooking frequency. Females are more prone to cooking, as well as people who live with a partner or have children in care (51). The results from our study are consistent with these determinants where 56.1% of female and 44.3% of male respondents cooked more during the confinement. Those respondents with children in care were also more prone to increasing their cooking frequency. Another determinant for cooking is leisure time, that is, people working longer hours indulge less in cooking (51). Due to the general confinement, people might be more prone to increase their cooking frequency because they were unable to go to restaurants and were not working or had a home office. An increase in cooking frequency and staying at home may have a beneficial impact on children and adolescents with a result of increasing their cooking skills (52). Moreover, during the confinement, people were advised not to go to the grocery store every day, which may have a positive influence resulting in healthier diet and lower odds of being obese due to imposed meal planning (53). While the motivation for introducing new lifestyle behaviors, such as cooking more and meal planning, resides in the fear of getting infected, this might be a trigger for habit formation (54, 55).

It is important to emphasize how cooking more frequently influences other dietary behaviors. It is suggested that eating more of home-cooked meals positively influences dietary behaviors, such as having greater adherence to the MedDiet, as well as a higher probability of having normal BMI value and body fat percentage (29). Furthermore, people with greater cooking skills also had higher weekly vegetable consumption and lower consumption of “unhealthier” food groups (56, 57). Our results indicate that the respondents who increased their cooking frequency during the COVID-19 confinement preferred olive oil as the main source of added fat. With an increase in cooking, respondents also significantly increased their vegetable intake, that is, those who cooked more had 2.24 higher odds of an increase in vegetable consumption during the confinement in comparison with the respondents who cooked the same as before or less. An increase in legume, fish and seafood, as well as homemade pastry consumption was also noted in the respondents who increased their cooking frequency during the confinement. These beneficial changes in dietary behavior reported by the respondents who cooked more during the confinement indicate that the higher cooking frequency might result in an overall greater diet quality. This result is confirmed by Wolfson et al. (58), whereas cooking more frequently was associated with a higher Healthy Eating Index.

This study has many strengths in providing insight into dietary behaviors under a new and challenging situation during the COVID-19 confinement policy in Croatia. The use of an online questionnaire proved itself as a useful tool in providing a relatively large number of respondents, which would be demanding to obtain by face-to-face interviews due to regulations for social distancing and general confinement. The COVIDiet questionnaire provided a great amount of information about dietary and lifestyle behaviors during the confinement.

In spite of the numerous strengths, some limitations must be mentioned. Due to web-based voluntary sampling, the responses resulted in some selection bias. Although an online questionnaire provided a large number of respondents, they were predominantly female (80.5%), 20–50 years old (82.1%), living in family homes (82.5%) in the continental part of Croatia (81.9%). It should be highlighted that the disproportion between continental and coastal parts of Croatia is more pronounced due to a large number of respondents residing in Zagreb, the capital city of Croatia (42.9%). The same selection bias was found for the respondents in the oldest age group, those living in a student's residence, as well as those with primary education level, where the proportion of these respondents overall was <1%. This might be because of the lower ability to use smart technologies for older people and those with primary education. Moreover, it should be emphasized that the respondents were untrained and were not able to ask for explanations for any doubts regarding questions. This might have resulted in under-/overestimation of actual food proportion intakes. Also, BMI was declared by participants and, therefore, should be treated only as a rough estimate of the exact number.

## Conclusion

COVID-19 is one of the greatest public health threats the world has been faced with, while nutritional status may have an important role in disease severity and clinical outcome. The most prevalently advised dietary pattern during this challenging time is the Mediterranean diet, well-known for its anti-inflammatory and immunomodulatory properties. According to our findings, Croatian adults exhibited medium adherence to the MedDiet during the COVID-19 confinement. Furthermore, higher adherence and higher eagerness to increase physical activity was associated with lower BMI values (<25 kg/m^2^). Regarding the dietary and lifestyle behavior aspects during the crisis, no change among the majority of respondents, in almost all of them, was found. An exception was the cooking frequency, where 53.8% of the respondents increased cooking incidence during the confinement. Those who cooked more also displayed an increase in vegetables, legumes, as well as fish and seafood consumption. These results suggest that cooking frequency could be positively associated with an overall dietary quality, which is of utmost importance in these demanding times.

## Data Availability Statement

The datasets presented in this article are not readily available because data has not been uploaded to publicly accessible repository. Requests to access the datasets should be directed to JR, jresetar@student.pharma.hr.

## Ethics Statement

The studies involving human participants were reviewed and approved by Research Ethics Committee of the University of Granada (1526/CEIH/2020). Written informed consent for participation was not required for this study in accordance with the national legislation and the institutional requirements.

## Author Contributions

CR-P and MR-L conceptualized and designed the study. IP, DV, and ZS were responsible for the conduction of the study. JK analyzed the data. DP and JG interpreted the data. DP, JR, and JG prepared the draft of the manuscript. All authors conducted critical analyses and then finally approved the manuscript. All authors have read and agreed to the published version of the manuscript.

## Conflict of Interest

The authors declare that the research was conducted in the absence of any commercial or financial relationships that could be construed as a potential conflict of interest.
