# Supplementary material for: Cooking at Home and Adherence to the Mediterranean Diet During the COVID-19 Confinement: The Experience From the Croatian COVIDiet Study
Source: Front Nutr. 2021 Mar 31;8:617721. doi: 10.3389/fnut.2021.617721 (PMC8044460; doi:10.3389/fnut.2021.617721)
Supplement: Supplementary file 1 [file Data_Sheet_1.docx]

Supplementary Material

**Table S1.** Changes in dietary behavior and life styles by the BMI values of Croatian respondents during the COVID-19 confinement.

|  | BMI groups | | | |  | | p-value^1^ |
| --- | --- | --- | --- | --- | --- | --- | --- |
|  | **< 25 kg/m^2^** | | **> 25 kg/m^2^** | | **All** | |  |
|  | N1=2869 | % | N2=1412 | % | N=4281 | % |  |
| Cooking more often | | | | | | | 0.111 |
| More | 1574 | 54.9 | 728 | 51.6 | 2302 | 53.8 |  |
| Less | 65 | 2.3 | 31 | 2.2 | 96 | 2.2 |  |
| As usual | 1230 | 42.9 | 653 | 46.2 | 1883 | 44.0 |  |
| Fried foods consumed | | | | | | | 0.126 |
| Higher | 175 | 6.1 | 109 | 7.7 | 284 | 6.6 |  |
| Lower | 690 | 24.1 | 341 | 24.2 | 1031 | 24.1 |  |
| As usual | 2004 | 69.9 | 962 | 68.1 | 2966 | 69.3 |  |
| Frequency of fried food consumed(times/week) | | | | | | | <0.001 |
| < 1 | 1113 | 38.8 | 445 | 31.5 | 1558 | 36.4 |  |
| 1-3 | 1457 | 50.8 | 817 | 57.9 | 2274 | 53.1 |  |
| 4-6 | 104 | 3.6 | 65 | 4.6 | 169 | 3.9 |  |
| > 7 | 7 | 0.2 | 6 | 0.4 | 13 | 0.3 |  |
| Never | 188 | 6.6 | 79 | 5.6 | 267 | 6.2 |  |
| Type of oil used for frying | | | | | | | 0.353 |
| Olive oil | 674 | 23.5 | 304 | 21.5 | 978 | 22.8 |  |
| Sunflower oil | 1818 | 63.4 | 916 | 64.9 | 2734 | 63.9 |  |
| Other | 377 | 13.1 | 192 | 13.6 | 569 | 13.3 |  |
| Frequency of snacking | | | | | | | <0.001 |
| Higher | 992 | 34.6 | 460 | 32.6 | 1452 | 33.9 |  |
| Lower | 292 | 10.2 | 201 | 14.2 | 493 | 11.5 |  |
| As usual | 1585 | 55.2 | 751 | 53.2 | 2336 | 54.6 |  |
| Frequency of fast-food consumed | | | | | | | 0.928 |
| Higher | 66 | 2.3 | 35 | 2.5 | 101 | 2.4 |  |
| Lower | 1553 | 54.1 | 766 | 54.2 | 2319 | 54.2 |  |
| As usual | 1250 | 43.6 | 611 | 43.3 | 1861 | 43.5 |  |
| Ate more | | | | | | | 0.401 |
| Yes | 1225 | 42.7 | 622 | 44.1 | 1847 | 43.1 |  |
| No | 1644 | 57.3 | 790 | 55.9 | 2434 | 56.9 |  |
| Modification of physical activity | | | | | | | <0.001 |
| It has increased | 899 | 31.3 | 331 | 23.4 | 1230 | 28.7 |  |
| It has decreased | 1058 | 36.9 | 523 | 37.0 | 1581 | 36.9 |  |
| It remains as usual | 751 | 26.2 | 415 | 29.4 | 1166 | 27.2 |  |
| No physical activity | 161 | 5.6 | 143 | 10.1 | 304 | 7.1 |  |
| Weight gain | | | | | | | <0.001 |
| Yes | 488 | 17.0 | 383 | 27.1 | 871 | 20.3 |  |
| No | 1740 | 60.6 | 723 | 51.2 | 2463 | 57.5 |  |
| I do not know | 641 | 22.3 | 306 | 21.7 | 947 | 22.1 |  |

^1^ Differences between the three Mediterranean diet adherence groups were evaluated by the Chi-squared test.

**Table S2.**Associations between respondents’ BMI values and the changes in dietary behavior of Croatian respondents during the COVID-19 confinement.

|  | BMI < 25 kg/m^2^ | | BMI > 25 kg/m^2^ | | All | | p-value^1^ | Crude^2^ | | Model 1^3^ | | Model 2^4^ | |
| --- | --- | --- | --- | --- | --- | --- | --- | --- | --- | --- | --- | --- | --- |
|  | N=1979 | % | N=2302 | % | N=4281 | % |  | OR | 95%CI | OR | 95%CI | OR | 95%CI |
| VEGETABLE CONSUMEd | | | | | | | 0.810 |  |  |  |  |  |  |
| Higher | 613 | 21.4 | 293 | 20.8 | 906 | 21.2 |  | 1.06 | [0.88;1.27] | 1.13 | [0.92;1.38] | 1.12 | [0.92;1.37] |
| Lower | 245 | 8.5 | 140 | 9.9 | 385 | 9.0 |  | 1.00 | [0.77;1.29] | 0.88 | [0.66;1.17] | 0.88 | [0.66;1.18] |
| As usual | 2011 | 70.1 | 979 | 69.3 | 2990 | 69.8 |  | Ref. |  | Ref. |  | Ref. |  |
| FRUIT CONSUMEd | | | | | | | 0.145 |  |  |  |  |  |  |
| Higher | 649 | 22.6 | 278 | 19.7 | 927 | 21.7 |  | 0.87 | [0.72;1.04] | 0.95 | [0.78;1.15] | 0.95 | [0.77;1.15] |
| Lower | 255 | 8.9 | 159 | 11.3 | 414 | 9.7 |  | 1.12 | [0.88;1.44] | 1.24 | [0.94;1.62] | 1.21 | [0.93;1.60] |
| As usual | 1965 | 68.5 | 975 | 69.1 | 2940 | 68.7 |  | Ref. |  | Ref. |  | Ref. |  |
| RED MEAT CONSUMEd | | | | | | | 0.923 |  |  |  |  |  |  |
| Higher | 257 | 9.0 | 136 | 9.6 | 393 | 9.2 |  | 1.03 | [0.81;1.32] | 1.11 | [0.85;1.45] | 1.12 | [0.85;1.48] |
| Lower | 620 | 21.6 | 316 | 22.4 | 936 | 21.9 |  | 0.98 | [0.82;1.17] | 0.94 | [0.78;1.15] | 0.96 | [0.79;1.17] |
| As usual | 1992 | 69.4 | 960 | 68.0 | 2952 | 69.0 |  | Ref. |  | Ref. |  | Ref. |  |
| CARBONATED AND/OR SUGARY BEVERAGES CONSUMEd | | | | | | | 0.068 |  |  |  |  |  |  |
| Higher | 154 | 5.4 | 81 | 5.7 | 235 | 5.5 |  | 1.10 | [0.81;1.49] | 1.33 | [0.95;1.85] | 1.25 | [0.90;1.76] |
| Lower | 699 | 24.4 | 397 | 28.1 | 1096 | 25.6 |  | **1.22** | **[1.03;1.45]** | **1.21** | **[1.01;1.46]** | 1.20 | [0.99;1.45] |
| As usual | 2016 | 70.3 | 934 | 66.2 | 2950 | 68.9 |  | Ref. |  | Ref. |  | Ref. |  |
| LEGUMES CONSUMEd | | | | | | | 0.824 |  |  |  |  |  |  |
| Higher | 323 | 11.3 | 143 | 10.1 | 466 | 10.9 |  | 0.95 | [0.76;1.19] | 0.99 | [0.78;1.27] | 1.03 | [0.80;1.31] |
| Lower | 162 | 5.7 | 99 | 7.0 | 261 | 6.1 |  | 1.06 | [0.79;1.41] | 1.02 | [0.74;1.39] | 1.05 | [0.76;1.44] |
| As usual | 2384 | 83.1 | 1170 | 82.9 | 3554 | 83.0 |  | Ref. |  | Ref. |  | Ref. |  |
| FISH-SEAFOOD CONSUMEd | | | | | | | 0.029 |  |  |  |  |  |  |
| Higher | 386 | 13.5 | 149 | 10.6 | 535 | 12.5 |  | 0.75 | [0.61;0.93] | 0.80 | [0.63;1.02] | 0.83 | [0.66;1.06] |
| Lower | 428 | 14.9 | 224 | 15.9 | 652 | 15.2 |  | 0.89 | [0.73;1.08] | 0.83 | [0.67;1.03] | 0.87 | [0.70;1.09] |
| As usual | 2055 | 71.6 | 1039 | 73.6 | 3094 | 72.3 |  | Ref. |  | Ref. |  | Ref. |  |
| COMMERCIAL PASTRIES CONSUMEd | | | | | | | 0.037 |  |  |  |  |  |  |
| Higher | 627 | 21.9 | 295 | 20.9 | 922 | 21.5 |  | 1.07 | [0.88;1.29] | 1.15 | [0.93;1.42] | 1.17 | [0.94;1.44] |
| Lower | 506 | 17.6 | 314 | 22.2 | 820 | 19.2 |  | **1.31** | **[1.07;1.60]** | **1.38** | **[1.11;1.73]** | **1.41** | **[1.12;1.76]** |
| As usual | 1736 | 60.5 | 803 | 56.9 | 2539 | 59.3 |  | Ref. |  | Ref. |  | Ref. |  |
| HOMEMADE PASTRIES CONSUMEd | | | | | | | <0.001 |  |  |  |  |  |  |
| Higher | 1032 | 36.0 | 403 | 28.5 | 1435 | 33.5 |  | 0.67 | [0.57;0.80] | 0.85 | [0.70;1.02] | 0.85 | [0.70;1.02] |
| Lower | 315 | 11.0 | 229 | 16.2 | 544 | 12.7 |  | **1.27** | **[1.01;1.60]** | **1.23** | **[0.96;1.59]** | **1.25** | **[0.97;1.61]** |
| As usual | 1522 | 53.1 | 780 | 55.2 | 2302 | 53.8 |  | Ref. |  | Ref. |  | Ref. |  |
| ALCOHOLIC BEVERAGE CONSUMEd | | | | | | | <0.001 |  |  |  |  |  |  |
| Higher | 296 | 10.3 | 135 | 9.6 | 431 | 10.1 |  | 0.77 | [0.61;0.97] | 0.70 | [0.54;0.89] | 0.73 | [0.57;0.94] |
| Lower | 840 | 29.3 | 347 | 24.6 | 1187 | 27.7 |  | 0.69 | [0.58;0.81] | 0.75 | [0.63;0.91] | 0.77 | [0.64;0.92] |
| As usual | 1733 | 60.4 | 930 | 65.9 | 2663 | 62.2 |  | Ref. |  | Ref. |  | Ref. |  |
| FRIED FOODS CONSUMEd | | | | | | | 0.425 |  |  |  |  |  |  |
| Higher | 175 | 6.1 | 109 | 7.7 | 284 | 6.6 |  | 1.18 | [0.89;1.57] | 1.13 | [0.83;1.54] | 1.10 | [0.80;1.50] |
| Lower | 690 | 24.1 | 341 | 24.2 | 1031 | 24.1 |  | 0.96 | [0.81;1.15] | 0.93 | [0.77;1.12] | 0.93 | [0.77;1.13] |
| As usual | 2004 | 69.9 | 962 | 68.1 | 2966 | 69.3 |  | Ref. |  | Ref. |  | Ref. |  |
| FREQUENCY OF SNACKING | | | | | | | 0.002 |  |  |  |  |  |  |
| Higher | 992 | 34.6 | 460 | 32.6 | 1452 | 33.9 |  | 0.90 | [0.77;1.06] | 1.06 | [0.89;1.27] | 1.08 | [0.91;1.30] |
| Lower | 292 | 10.2 | 201 | 14.2 | 493 | 11.5 |  | **1.37** | **[1.10;1.69]** | **1.61** | **[1.27;2.03]** | **1.67** | **[1.31;2.11]** |
| As usual | 1585 | 55.3 | 751 | 53.2 | 2336 | 54.6 |  | Ref. |  | Ref. |  | Ref. |  |
| FREQUENCY OF FAST-FOOD CONSUMEd | | | | | | | 0.829 |  |  |  |  |  |  |
| Higher | 66 | 2.3 | 35 | 2.5 | 101 | 2.4 |  | 0.96 | [0.61;1.52] | 1.29 | [0.78;2.13] | 1.40 | [0.84;2.32] |
| Lower | 1553 | 54.1 | 766 | 54.3 | 2319 | 54.2 |  | 1.04 | [0.90;1.21] | **1.19** | **[1.01;1.40]** | 1.18 | [1.00;1.39] |
| As usual | 1250 | 43.6 | 611 | 43.3 | 1861 | 43.5 |  | Ref. |  | Ref. |  | Ref. |  |
| WEIGHT GAIN | | | | | | | <0.001 |  |  |  |  |  |  |
| Yes | 488 | 17.0 | 383 | 27.1 | 871 | 20.4 |  | **1.76** | **[1.45;2.15]** | **1.50** | **[1.21;1.86]** | **1.53** | **[1.23;1.90]** |
| No | 1740 | 60.7 | 723 | 51.2 | 2463 | 57.5 |  | 0.77 | [0.65;0.92] | 0.65 | [0.54;0.79] | 0.66 | [0.55;0.80] |
| I do not know | 641 | 22.3 | 306 | 21.7 | 947 | 22.1 |  | Ref. |  | Ref. |  | Ref. |  |

^1^ Differences between the groups were evaluated by the Chi-squared test; ^2^ Crude model: unadjusted for any variable; ^3^ Model 1: multivariate adjusted model for gender (women, men and other), age groups (< 20, 20 – 35 y, 36 – 50 y, 51 – 65 y and > 65 y) and regions (continental and coastal). ^4^ Model 2: multivariate adjusted model for gender, age groups, regions, residence (alone, family home, shared flat and student’s residence), education level (university, postgraduate, professional and primary) and physical activity (higher, lower, similar, never). Odds ratios (ORs) and corresponding 95% confidence intervals were estimated for all models. In addition, statistically significant ORs are highlighted in bold.

**Table S3.**Outlines of questionnaire responds by the change of cooking frequencyof Croatian respondents during the COVID-19 confinement.

| Respondents’ characteristics | cooking more | | cooking less | | cooking as usual | | All | | p-value^2^ |
| --- | --- | --- | --- | --- | --- | --- | --- | --- | --- |
|  | N1=2302 | % | N2=96 | % | N3=1883 | % | N=4281 | % |  |
| Gender | | | | | | | | | <0.001 |
| Male | 367 | 15.9 | 22 | 22.9 | 440 | 23.4 | 829 | 19.4 |  |
| Female | 1933 | 84.0 | 72 | 75.0 | 1439 | 76.4 | 3444 | 80.4 |  |
| Other | 2 | 0.1 | 2 | 2.1 | 4 | 0.2 | 8 | 0.2 |  |
| Place of residence | | | | | | | | | <0.001 |
| Family home | 1848 | 52.3 | 67 | 1.9 | 1617 | 45.8 | 3532 | 82.5 |  |
| Shared flat | 162 | 64.0 | 13 | 5.1 | 78 | 30.8 | 253 | 5.9 |  |
| Alone | 277 | 59.3 | 16 | 3.4 | 174 | 37.3 | 467 | 10.9 |  |
| Student's residence | 15 | 51.7 | 0 | 0.0 | 14 | 48.3 | 29 | 0.7 |  |
| Region by Areas^1^ | | | | | | | | | 0.005 |
| Continental part | 1922 | 54.8 | 72 | 2.1 | 1511 | 43.1 | 3505 | 81.9 |  |
| Coastal part | 380 | 49.0 | 24 | 3.1 | 372 | 47.9 | 776 | 18.1 |  |
| Children in care | | | | | | | | | <0.001 |
| Yes | 985 | 57.0 | 17 | 1.0 | 727 | 42.0 | 1729 | 40.4 |  |
| No | 1317 | 51.6 | 79 | 3.1 | 1156 | 45.3 | 2552 | 59.6 |  |
| Education level | | | | | | | | | <0.001 |
| University | 1400 | 59.2 | 36 | 1.5 | 928 | 39.3 | 2364 | 55.2 |  |
| Postgraduate | 432 | 52.9 | 26 | 3.2 | 358 | 43.9 | 816 | 19.1 |  |
| Professional | 461 | 43.2 | 33 | 3.1 | 572 | 53.7 | 1066 | 24.9 |  |
| Primary | 9 | 25.7 | 1 | 2.9 | 25 | 71.4 | 35 | 0.8 |  |
| Age | | | | | | | | | <0.001 |
| < 20 | 81 | 43.5 | 15 | 8.1 | 90 | 48.4 | 186 | 4.3 |  |
| 20 - 35 | 1064 | 56.2 | 48 | 2.5 | 780 | 41.2 | 1892 | 44.2 |  |
| 36 - 50 | 941 | 58.0 | 18 | 1.1 | 663 | 40.9 | 1622 | 37.9 |  |
| 51 - 65 | 212 | 39.2 | 12 | 2.2 | 317 | 58.6 | 541 | 12.6 |  |
| > 65 | 4 | 10.0 | 3 | 7.5 | 33 | 82.5 | 40 | 0.9 |  |
| BMI | | | | | | | | | 0.315 |
| Underweight | 118 | 51.5 | 4 | 1.7 | 107 | 46.7 | 229 | 5.3 |  |
| Normal | 1631 | 55.0 | 67 | 2.3 | 1266 | 42.7 | 2964 | 69.2 |  |
| Overweight | 360 | 50.6 | 15 | 2.1 | 336 | 47.3 | 711 | 16.6 |  |
| Obesity | 193 | 51.2 | 10 | 2.7 | 174 | 46.2 | 377 | 8.8 |  |
| MEDAS | | | | | | | | | <0.001 |
| Low | 641 | 48.8 | 35 | 2.7 | 639 | 48.6 | 1315 | 30.7 |  |
| Medium | 1573 | 56.1 | 61 | 2.2 | 1172 | 41.7 | 2806 | 65.6 |  |
| High | 88 | 55.0 | 0 | 0.0 | 72 | 45.0 | 160 | 3.7 |  |

^1^ Official EU division areas for Croatia – NUTS 2 (Official journal, 2012); ^2^ Differences between the three Mediterranean diet adherence groups were evaluated by the Chi-squared test.

**Table S4.**Eating behavior by the change of cooking frequencyof Croatian respondents during the COVID-19 confinement.

|  | Cooking more | | Cooking less | | Cooking as usual | | All | | p-value^1^ | MEDAS |
| --- | --- | --- | --- | --- | --- | --- | --- | --- | --- | --- |
|  | N1=2302 | % | N2=96 | % | N3=1883 | % | N=4281 | % |  |  |
| Olive oil consumed(tablespoons (13.5 g)/day) | | | | | | | | | <0.001 | (i) |
| > 4 | 480 | 20.9 | 11 | 11.5 | 377 | 20.0 | 868 | 20.3 |  |  |
| 2-3.9 | 847 | 36.8 | 30 | 31.3 | 583 | 31.0 | 1460 | 34.1 |  |  |
| 0-1.9 | 975 | 42.4 | 55 | 57.3 | 923 | 49.0 | 1953 | 45.6 |  |  |
| Vegetables consumed (servings (200 g)/day) | | | | | | | | | 0.076 | (ii) |
| > 2 | 492 | 21.4 | 21 | 21.9 | 434 | 23.0 | 947 | 22.1 |  |  |
| 1-1.9 | 1419 | 61.6 | 50 | 52.1 | 1107 | 58.8 | 2576 | 60.2 |  |  |
| 0-0.9 | 391 | 17.0 | 25 | 26.0 | 342 | 18.2 | 758 | 17.7 |  |  |
| Fruit consumed(pieces/day) | | | | | | | | | 0.402 | (iii) |
| > 3 | 374 | 16.2 | 16 | 16.7 | 307 | 16.3 | 697 | 16.3 |  |  |
| 1-2.9 | 1314 | 57.1 | 46 | 47.9 | 1072 | 56.9 | 2432 | 56.8 |  |  |
| 0-0.9 | 614 | 26.7 | 34 | 35.4 | 504 | 26.8 | 1152 | 26.9 |  |  |
| Red meat consumed(servings (100-150 g)/day) | | | | | | | | | 0.591 | (iv) |
| > 1 | 685 | 29.8 | 33 | 34.4 | 573 | 30.4 | 1291 | 30.2 |  |  |
| 0-0.9 | 1617 | 70.2 | 63 | 65.6 | 1310 | 69.6 | 2990 | 69.8 |  |  |
| Butter, margarine or cream consumed (servings(12 g)/day) | | | | | | | | | 0.209 | (v) |
| > 1 | 417 | 18.1 | 18 | 18.8 | 303 | 16.1 | 738 | 17.2 |  |  |
| 0-0.9 | 1885 | 81.9 | 78 | 81.3 | 1580 | 83.9 | 3543 | 82.8 |  |  |
| Carbonated and/or sugary beverages consumed (times/day) | | | | | | | | | 0.120 | (vi) |
| > 1 | 179 | 7.8 | 13 | 13.5 | 157 | 8.3 | 349 | 8.2 |  |  |
| 0-0.9 | 2123 | 92.2 | 83 | 86.5 | 1726 | 91.7 | 3932 | 91.8 |  |  |
| Wine consumed(cups (100 mL)/week) | | | | | | | | | <0.001 | (vii) |
| > 7 | 80 | 3.5 | 5 | 5.2 | 42 | 2.2 | 127 | 3.0 |  |  |
| 3-6.9 | 198 | 8.6 | 13 | 13.5 | 121 | 6.4 | 332 | 7.8 |  |  |
| 0-2.9 | 1167 | 50.7 | 34 | 35.4 | 872 | 46.3 | 2073 | 48.4 |  |  |
| I never drink wine | 857 | 37.2 | 44 | 45.8 | 848 | 45.0 | 1749 | 40.9 |  |  |
| Legumes consumed(servings (150 g)/week) | | | | | | | | | 0.139 | (viii) |
| > 3 | 245 | 10.6 | 13 | 13.5 | 198 | 10.5 | 456 | 10.7 |  |  |
| 1-2.9 | 1289 | 56.0 | 41 | 42.7 | 1061 | 56.3 | 2391 | 55.9 |  |  |
| 0-0.9 | 768 | 33.4 | 42 | 43.8 | 624 | 33.1 | 1434 | 33.5 |  |  |
| Fish-seafood consumed(servings (100-150 g for fish or 200 g for seafood)/week) | | | | | | | | | 0.888 | (ix) |
| > 3 | 113 | 4.9 | 6 | 6.3 | 101 | 5.4 | 220 | 5.1 |  |  |
| 1-2.9 | 1095 | 47.6 | 42 | 43.8 | 882 | 46.8 | 2019 | 47.2 |  |  |
| 0-0.9 | 1094 | 47.5 | 48 | 50.0 | 900 | 47.8 | 2042 | 47.7 |  |  |
| Commercial pastries consumed(times/week) | | | | | | | | | 0.008 | (x) |
| > 2 | 940 | 40.8 | 40 | 41.7 | 682 | 36.2 | 1662 | 38.8 |  |  |
| 0-1.9 | 1362 | 59.2 | 56 | 58.3 | 1201 | 63.8 | 2619 | 61.2 |  |  |
| Nuts consumed(servings (30 g)/week) | | | | | | | | | <0.001 | (xi) |
| 1-2.9 | 1409 | 61.2 | 41 | 42.7 | 1054 | 56.0 | 2504 | 58.5 |  |  |
| 0-0.9 | 893 | 38.8 | 55 | 57.3 | 829 | 44.0 | 1777 | 41.5 |  |  |
| vegetables, pasta, rice cooked with olive oil(servings/week) | | | | | | | | | <0.001 | (xii) |
| > 2 | 1430 | 62.1 | 51 | 53.1 | 1064 | 56.5 | 2545 | 59.4 |  |  |
| 1-1.9 | 648 | 28.1 | 28 | 29.2 | 576 | 30.6 | 1252 | 29.2 |  |  |
| 0-0.9 | 224 | 9.7 | 17 | 17.7 | 243 | 12.9 | 484 | 11.3 |  |  |
| PREFERRED OLIVE OIL IN COOKING | | | | | | | | | <0.001 | (xiii) |
| Yes | 1178 | 51.2 | 34 | 35.4 | 812 | 43.1 | 2024 | 47.3 |  |  |
| No | 1124 | 48.8 | 62 | 64.6 | 1071 | 56.9 | 2257 | 52.7 |  |  |
| PreferRed white meat instead of red meat | | | | | | | | | 0.263 | (xiv) |
| Yes | 1634 | 71.0 | 62 | 64.6 | 1307 | 69.4 | 3003 | 70.1 |  |  |
| No | 668 | 29.0 | 34 | 35.4 | 576 | 30.6 | 1278 | 29.9 |  |  |

^1^ Differences between the three Mediterranean diet adherence groups were evaluated by the Chi-squared test.

**Table S5.**Associations between the change in cooking practice and eating behavior of Croatian respondents during the COVID-19 confinement.

|  | As usual or less | | Cooking more | | p-value^1^ | Crude^2^ | | Model 1^3^ | | Model 2^4^ | |
| --- | --- | --- | --- | --- | --- | --- | --- | --- | --- | --- | --- |
|  | N=1979 | % | N=2302 | % |  | OR | 95% CI | OR | 95% CI | OR | 95% CI |
| OLIVE OIL CONSUMEd(tablespoons (13.5 g)/day) | | | | | <0.001 |  |  |  |  |  |  |
| > 4 | 388 | 19.6 | 480 | 20.9 |  | Ref. |  | Ref. |  | Ref. |  |
| 2-3.9 | 613 | 31.0 | 847 | 36.8 |  | 1.00 | [0.83;1.21] | 1.01 | [0.83;1.23] | 1.03 | [0.84;1.26] |
| 0-1.9 | 978 | 49.4 | 975 | 42.4 |  | 1.17 | [1.00;1.36] | **1.19** | **[1.02;1.40]** | 1.18 | [1.00;1.39] |
| VEGETABLES CONSUMEd(servings (200 g)/day) | | | | | 0.105 |  |  |  |  |  |  |
| > 2 | 455 | 23.0 | 492 | 21.4 |  | Ref. |  | Ref. |  | Ref. |  |
| 1-1.9 | 1157 | 58.5 | 1419 | 61.6 |  | 0.94 | [0.76;1.17] | 0.87 | [0.70;1.08] | 0.89 | [0.72;1.12] |
| 0-0.9 | 367 | 18.5 | 391 | 17.0 |  | 1.09 | [0.92;1.29] | 1.05 | [0.88;1.25] | 1.06 | [0.89;1.27] |
| FRUIT CONSUMEd(pieces/day) | | | | | 0.917 |  |  |  |  |  |  |
| > 3 | 323 | 16.3 | 374 | 16.2 |  | Ref. |  | Ref. |  | Ref. |  |
| 1-2.9 | 1118 | 56.5 | 1314 | 57.1 |  | 0.98 | [0.80;1.20] | 1.06 | [0.86;1.31] | 1.05 | [0.85;1.29] |
| 0-0.9 | 538 | 27.2 | 614 | 26.7 |  | 0.98 | [0.85;1.14] | 1.02 | [0.88;1.19] | 1.00 | [0.86;1.17] |
| RED MEAT CONSUMEd(servings (100-150 g)/day) | | | | | 0.539 |  |  |  |  |  |  |
| > 1 | 606 | 30.6 | 685 | 29.8 |  | Ref. |  | Ref. |  | Ref. |  |
| 0-0.9 | 1373 | 69.4 | 1617 | 70.2 |  | 1.02 | [0.88;1.17] | 1.06 | [0.92;1.22] | 1.15 | [0.99;1.33] |
| BUTTER, MARGARINE OR CREAM CONSUMEd(servings (12 g)/day) | | | | | 0.102 |  |  |  |  |  |  |
| > 1 | 321 | 16.2 | 417 | 18.1 |  | Ref. |  | Ref. |  | Ref. |  |
| 0-0.9 | 1658 | 83.8 | 1885 | 81.9 |  | 1.16 | [0.99;1.37] | **1.19** | **[1.01;1.41]** | **1.21** | **[1.02;1.44]** |
| CARBONATED AND/OR SUGARY BEVERAGES CONSUMEd(times/day) | | | | | 0.332 |  |  |  |  |  |  |
| > 1 | 170 | 8.6 | 179 | 7.8 |  | Ref. |  | Ref. |  | Ref. |  |
| 0-0.9 | 1809 | 91.4 | 2123 | 92.2 |  | 0.90 | [0.72;1.14] | 0.94 | [0.75;1.19] | 0.96 | [0.76;1.22] |
| WINE CONSUMEd(cups (100 mL)/week) | | | | | <0.001 |  |  |  |  |  |  |
| > 7 | 47 | 2.4 | 80 | 3.5 |  | Ref. |  | Ref. |  | Ref. |  |
| 3-6.9 | 134 | 6.8 | 198 | 8.6 |  | **1.79** | **[1.22;2.63]** | **2.03** | **[1.36;3.03]** | **1.96** | **[1.31;2.94]** |
| 0-2.9 | 906 | 45.8 | 1167 | 50.7 |  | **1.46** | **[1.15;1.87]** | **1.47** | **[1.15;1.89]** | **1.36** | **[1.06;1.76]** |
| I never drink wine | 892 | 45.1 | 857 | 37.2 |  | **1.31** | **[1.15;1.49]** | **1.35** | **[1.18;1.54]** | **1.27** | **[1.11;1.46]** |
| LEGUMES CONSUMEd(servings (150 g)/week) | | | | | 0.977 |  |  |  |  |  |  |
| > 3 | 211 | 10.7 | 245 | 10.6 |  | Ref. |  | Ref. |  | Ref. |  |
| 1-2.9 | 1102 | 55.7 | 1289 | 56.0 |  | 0.92 | [0.73;1.15] | 0.88 | [0.70;1.11] | 0.91 | [0.72;1.15] |
| 0-0.9 | 666 | 33.7 | 768 | 33.4 |  | 0.94 | [0.81;1.08] | 0.91 | [0.79;1.06] | 0.94 | [0.81;1.09] |
| FISH-SEAFOOD CONSUMEd(servings (100-150 g for fish or 200 g for seafood)/week) | | | | | 0.698 |  |  |  |  |  |  |
| > 3 | 107 | 5.4 | 113 | 4.9 |  | Ref. |  | Ref. |  | Ref. |  |
| 1-2.9 | 924 | 46.7 | 1095 | 47.6 |  | 0.78 | [0.58;1.05] | 0.82 | [0.61;1.11] | 0.83 | [0.61;1.12] |
| 0-0.9 | 948 | 47.9 | 1094 | 47.5 |  | 0.92 | [0.80;1.04] | 0.93 | [0.82;1.07] | 0.94 | [0.82;1.07] |
| COMMERCIAL PASTRIES CONSUMEd(times/week) | | | | | 0.004 |  |  |  |  |  |  |
| > 2 | 722 | 36.5 | 940 | 40.8 |  | Ref. |  | Ref. |  | Ref. |  |
| 0-1.9 | 1257 | 63.5 | 1362 | 59.2 |  | **1.22** | **[1.06;1.39]** | **1.24** | **[1.08;1.42]** | **1.19** | **[1.04;1.37]** |
| NUTS CONSUMEd(servings (30 g)/week) | | | | | <0.001 |  |  |  |  |  |  |
| 1-2.9 | 1095 | 55.3 | 1409 | 61.2 |  | Ref. |  | Ref. |  | Ref. |  |
| 0-0.9 | 884 | 44.7 | 893 | 38.8 |  | 1.12 | [0.98;1.29] | 1.02 | [0.89;1.18] | 1.07 | [0.92;1.23] |
| vegetables, pasta, rice cooked in olive oil(times/week) | | | | | <0.001 |  |  |  |  |  |  |
| > 2 | 1115 | 56.3 | 1430 | 62.1 |  | Ref. |  | Ref. |  | Ref. |  |
| 1-1.9 | 604 | 30.5 | 648 | 28.1 |  | 1.15 | [0.93;1.42] | 1.17 | [0.94;1.46] | 1.19 | [0.95;1.49] |
| 0-0.9 | 260 | 13.1 | 224 | 9.7 |  | **1.23** | **[1.08;1.41]** | **1.16** | **[1.02;1.33]** | **1.17** | **[1.02;1.34]** |
| PREFERRED OLIVE OIL IN COOKING | | | | | <0.001 |  |  |  |  |  |  |
| Yes | 846 | 42.7 | 1178 | 51.2 |  | Ref. |  | Ref. |  | Ref. |  |
| No | 1133 | 57.3 | 1124 | 48.8 |  | **1.29** | **[1.11;1.49]** | **1.37** | **[1.18;1.59]** | **1.28** | **[1.09;1.49]** |
| PREFERRED WHITE MEAT INSTEAD OF RED MEAT | | | | | 0.198 |  |  |  |  |  |  |
| Yes | 1369 | 69.2 | 1634 | 71.0 |  | Ref. |  | Ref. |  | Ref. |  |
| No | 610 | 30.8 | 668 | 29.0 |  | **1.29** | **[1.05;1.58]** | **1.29** | **[1.05;1.60]** | **1.28** | **[1.04;1.59]** |

^1^ Differences between the groups were evaluated by the Chi-squared test; ^2^ Crude model: unadjusted for any variable; ^3^ Model 1: multivariant adjusted model for gender (women, men and other), age groups (< 20, 20 – 35 y, 36 – 50 y, 51 – 65 y and > 65 y) and regions (continental and coastal). ^4^ Model 2: multivariant adjusted model for gender, age groups, regions, residence (alone, family home, shared flat and student’s residence), education level (university, postgraduate, professional and primary) and physical activity (higher, lower, similar, never). Odds ratios (ORs) and corresponding 95% confidence intervals were estimated for all models. In addition, statistically significant ORs are highlighted in bold.
